# Evaluation of antitumor potential of an anti-glypican-1 monoclonal antibody in preclinical lung cancer models reveals a distinct mechanism of action

**DOI:** 10.37349/etat.2024.00238

**Published:** 2024-06-17

**Authors:** Minghua Li, Yanhong Wang, Xiaoyang Lin, Haiqiang Yang, Xiaolin Zhang, Yun Bai, Xiankun Li, Lulu Zhang, Feng Cheng, Chuanhai Cao, Qingyu Zhou

**Affiliations:** National Research Council (CNR), Italy; ^1^Department of Pharmaceutical Sciences, Taneja College of Pharmacy, University of South Florida, Tampa, FL 33612, USA; ^2^MegaNano Biotech Inc., Tampa, FL 33612, USA; ^3^Zhengzhou Molecular Diagnosis Engineering Technology Research Center, Zhengzhou 450001, Henan Province, China

**Keywords:** Glypican-1, therapeutic monoclonal antibody, non-small cell lung carcinoma, orthotopic lung tumor models, tumor associated fibroblasts

## Abstract

**Aim:**

The main objective of this study was to investigate the antitumor effect of a mouse anti-human glypican-1 (GPC1) monoclonal antibody (mAb) on non-small cell lung carcinoma (NSCLC) and associated molecular mechanisms.

**Methods:**

The anti-proliferative and anti-migratory activities of anti-GPC1 mAb were examined in A549 and H460 NSCLC cells and LL97A lung fibroblasts. The inhibitory effect of anti-GPC1 mAb on tumor growth was evaluated in an orthotopic lung tumor model.

**Results:**

The in vitro study showed that anti-GPC1 mAb profoundly inhibited the anchorage-independent growth of A549 and H460 NSCLC cells and exhibited relatively high cytotoxic activities towards LL97A lung fibroblasts, A549/LL97A and H460/LL97A coculture spheroids. Moreover, anti-GPC1 mAb significantly decreased the expression of phospho-Src (p-Src; Tyr416), p-Akt (Ser473) and β-catenin in the co-cultured LL97A lung fibroblasts, and the expression of phospho-mitogen-activated protein kinase kinase (p-MEK; Ser217/221) and phospho-90 kDa ribosomal s6 kinase (p-p90RSK; Ser380) in co-cultured A549 cells. When anti-GPC1 mAb was administered to tumor-bearing mice, the inhibitory effect of anti-GPC1 mAb on the orthotopic lung tumor growth was not statistically significant. Nonetheless, results of Western blot analysis showed significant decrease in the phosphorylation of fibroblast growth factor receptor 1 (FGFR1) at Tyr766, Src at Tyr416, extracellular signal-regulated kinase (ERK) at Thr202/Tyr204, 90 kDa ribosomal S6 kinase (RSK) at Ser380, glycogen synthase kinases 3α (GSK3α) at Ser21 and GSK3β at Ser9 in tumor tissues. These data implicate that anti-GPC1 mAb treatment impairs the interaction between tumor cells and tumor associated fibroblasts by attenuating the paracrine FGFR signal transduction.

**Conclusions:**

The relatively potent cytotoxicity of anti-GPC1 mAb in lung fibroblasts and its potential inhibitory effect on the paracrine FGFR signal transduction warrant further studies on the combined use of this mAb with targeted therapeutics to improve therapeutic outcomes in lung cancer.

## Introduction

Lung cancer is the second most common cancer among both male and female and the leading cause of cancer-related mortality in the United States [1]. Non-small cell lung carcinoma (NSCLC) accounts for approximately 85% of all lung cancer cases. Despite therapeutic advances [2], prognosis of metastatic NSCLC (stages IIIB and IV) remains extremely poor, with the median survival time being less than 13 months [3], indicating a pressing need for new treatment options with novel mechanisms of action for patients with NSCLC.

Glypican-1 (GPC1) is a member of the glypican family of cell-surface heparan sulphate proteoglycans (HSPGs) bound to cell membranes through a glycosylphosphatidylinositol (GPI) anchor [4]. It functions as a co-receptor/modulator for growth factors that are bound to heparin or heparan sulfate, including fibroblast growth factor 2 (FGF2) [5, 6], tumor growth factor β (TGFβ) [7], hepatocyte growth factor (HGF) [8], vascular endothelial growth factor (VEGF) [9], Hedgehogs (HHs) [10], and members of the Wingless-type (Wnt) protein family of secreted glycoproteins [11]. The modulatory effects of GPC1 on the activities of those growth factors have been linked to the enhanced cell adhesion, migration and lipoprotein metabolism [12, 13].

Given the aberrant GPC1 expression in solid tumors and its unique role in tumor progression, a number of studies have explored the diagnostic and prognostic value of GPC1 in certain tumor types, including pancreatic cancer [14, 15], breast cancer [16], prostate cancer [17], colorectal cancer [18, 19], glioblastomas [6, 20], esophageal squamous cell carcinoma (ESCC) [21] and NSCLC [22, 23]. Several studies have reported that GPC1-positive circulating exosomes in plasma might serve as a diagnostic marker for cancer detection [14, 24], while some have shown conflicting results [25–27]. The clinicopathological significance and diagnostic role of GPC1 in NSCLC remain obscure. One study showed that the GPC1 protein expression was detected by immunohistochemistry (IHC) in all 33 cases of mesotheliomas and 20 out of 21 cases of lung adenocarcinoma [22], whereas the IHC result obtained from another study showed that GPC1 was expressed in all 82 cases of epithelioid mesothelioma, but only 3 out of 97 cases of lung adenocarcinoma [23].

Several studies have examined the potential of GPC1 as a druggable target in the treatment of solid tumors. Stable transfection of MDA-MB-231 and MDA-MB-468 breast cancer cells with a GPC1 antisense construct markedly decreased GPC1 protein levels and the mitogenic response to heparin-binding (HB)-EGF, FGF2, heregulin α, heregulin β, and HGF [16]. Stable transfection of a full-length GPC1 antisense construct downregulated *GPC1* gene expression in Colo-357 pancreatic cancer cells, resulting in decreases in anchorage-dependent and -independent cell growth, TGFβ1-induced cell growth inhibition, nuclear translocation of phospho-Smad-2 and activity of plasminogen activator inhibitor-1 (PAI-1) promoter [28]. Treatment with 10 mg/kg of a chicken/mouse chimeric anti-human GPC1 monoclonal antibody (mAb) was found to suppress tumor growth via the antibody-dependent cell-mediated cytotoxicity (ADCC) and complement-dependent cytotoxicity (CDC) as well as inhibition of tumor angiogenesis in a GPC1-positive TE14 human ESCC xenograft model and a patient-derived tumor xenograft (PDX) model [29]. Overall, it has been demonstrated that reduction of GPC1 activity through molecular or pharmacological interventions results in suppression of growth factor induced tumor cell growth, suggesting the potential of GPC1 as a druggable therapeutic target [16, 28, 29].

mAb-based targeted therapy offers many benefits over conventional chemotherapy in terms of dosing frequency, potency, and specificity for the target antigen. In this study, the anti-tumor effect of an anti-GPC1 mAb was evaluated in A549 and H460 NSCLC cell lines and LL97A human lung fibroblasts using a variety of 2D and 3D monoculture and co-culture models and in an orthotopic A549 lung tumor xenograft model. The molecular response to anti-GPC1 mAb treatment in tumors was examined using Western blotting analysis. Results of the in vitro study revealed that LL97A lung fibroblasts, A549/LL97A and H460/LL97A co-culture spheroids were more sensitive to the cytotoxic effect of anti-GPC1 mAb as compared with A549 and H460 NSCLC cell monocultures. Results of the in vivo study showed that the anti-GPC1 mAb treatment was unable to inhibit A549 lung tumor growth significantly. Nonetheless, phosphorylation of FGFR1 at Tyr766 and Src at Tyr416 was significantly decreased in anti-GPC1 mAb treated A549 lung tumors.

## Materials and methods

### Cell lines and cell culture

Two human NSCLC cell lines, A549 [American Type Culture Collection (ATCC)^®^ CCL-185] and H460 (HTB-177^TM^), and a human lung fibroblast cell line, LL97A (CCL-191^TM^) were purchased from the ATCC (Manassas, VA, USA). The A549-Red-FLuc Bioware^®^ Brite cell line, which has been stably transduced with the red-shifted firefly luciferase gene from *Luciola italica* (Red-FLuc) was purchased from PerkinElmer Inc. (Waltham, WA, USA). Authentication of A549, H460, LL97A and A549-Red-Fluc cell lines was performed by IDEXX BioAnalytics to ensure that individual cell lines were correctly identified and free of contamination of non-human cells (Columbia, MO, USA). A549, A549-Red-Fluc, and LL97A cell lines were cultured in a mixture of Dulbecco’s Modified Eagle’s Medium (DMEM)/Ham’s F12 at a ratio of 1:1 (Corning Inc., Corning, NY, USA) supplemented with 10% heat-inactivated fetal bovine serum (FBS; Thermo Fisher Scientific, Waltham, MA, USA). The H460 cell line was cultured in RPMI-1640 medium (Corning Inc., Corning, NY, USA) supplemented with 10% heat-inactivated FBS. Penicillin (100 units/mL) and streptomycin (100 µg/mL) were added to the cell culture media to prevent contamination. Cells were maintained at 37℃ in a humidified atmosphere of 5% CO_2_ in air and tested for mycoplasma using the MycoAlert Mycoplasma Detection Kit (catalog no.: LT07-118, Lonza, Allendale, NJ, USA) periodically throughout the studies to ensure that all cells used in the study were free from mycoplasma contamination.

### Animals

Male athymic nude mice (Hsd: Athymic Nude-Foxn1nu; 5–6 weeks old) were purchased from Envigo (Indianapolis, IN, USA).

### Preparation of therapeutic anti-human GPC1 mouse monoclonal antibody

The therapeutic anti-human GPC1 mouse mAb was prepared by MegaNano Biotech Inc. (Tampa, FL, USA). In brief, human *GPC1* gene and fragments (two overlapping fragments with one from AA1−280 and the other one from AA240−559. Sequence information was provided in Supplemental materials) were cloned into PGEX-6p-1 expression vector and expressed in BL21 bacteria. Fusion proteins were cleaved from glutathione Sepharose 4B beads with PreScission protease (Sigma-Aldrich Inc., St. Louis, Mo, USA). For immunization and hybridoma generation, mouse splenocytes were isolated and fused with Sp2/0 mouse myeloma cells. Positive clones selected with human recombinant protein were amplified in large quantity. Antibodies were lyophilized after being isolated with protein A beads.

### MTT assay

The in vitro cytotoxic activity of anti-GPC1 mAb was determined in cultured A549 and H460 human NSCLC cells and LL97A human lung fibroblasts by an MTT assay as described previously with minor modifications [30]. A549 and H460 human NSCLC cells and LL97A lung fibroblasts were seeded in 96-well plates at a density of 4 × 10E3 cells/well and allowed to attach overnight. On the next day, culture media containing anti-GPC1 mAb (4.5–290 μg/mL) were added to appropriate wells. After the cells were treated for 72 h, an aliquot of 10 μL of 5 mg/mL MTT dissolved in phosphate-buffered saline (PBS) was added to each well, and cells were incubated at 37℃ for another 4 h. Finally, cells were lysed for 1 h with the addition of 100 μL of 100% DMSO. Plates were read at 570 nm on a SpectraMax 190 microplate reader equipped with SoftMax Pro software (Molecular Devices, Sunnyvale, CA, USA). The growth of treated cells was expressed as a percentage of vehicle control cultures. The concentration of anti-GPC1 mAb required for 50% inhibition of cell growth (i.e., IC_50_) as compared with the control cells was calculated by nonlinear fitting of the experimental data obtained from multiple independent experiments performed in quadruplicates using the GraphPad Prism 5.0 program (GraphPad Software, Inc. La Jolla, CA, USA).

### 3-Dimentional cytotoxicity assay

A549 and H460 monoculture spheroids and A549/LL97A and H460/LL97A coculture spheroids (in a tumor cell to fibroblast ratio of 1:1) were generated by seeding a total of 4,000 cells per well in 200 μL of complete culture medium on a Spheron Nunclon 96-well plate (Thermo Fisher Scientific, Waltham, MA, USA) followed by centrifugation at 400 × *g* for 10 min. After the spheroids were cultured for 24 h, various amounts of anti-GPC1 mAb were added to achieve the final concentrations of 40 μg/mL, 80 μg/mL, 120 μg/mL, 160 μg/mL and 200 μg/mL and incubated for 72 h. After the 72-hour treatment with the vehicle control and anti-GPC1 mAb, spheroid viability was measured by reading luminescence (Synergy Neo2 Hybrid Multi-Mode Reader; Biotek Instruments, Inc. Winooski, VT, USA) after 20 min incubation with CellTiter-Glo 3D reagent (50 µL/well; Promega Co. Madison, WI, USA) according to the manufacturer’s protocol.

### Soft agar colony formation assay

The inhibitory effect of anti-GPC1 mAb on the anchorage-independent growth of A549 and H460 tumor cells was evaluated using a modified two-layer soft agar culture system [31]. For the bottom layer of agar, 0.3 mL of complete culture medium containing 0.5% noble agar (Thermo Fisher Scientific, Waltham, MA, USA) was added to each well of a 24-well plate. After the bottom layer was solidified, 2 × 10E3 A549 or H460 cells were suspended in 0.2 mL of the same culture medium containing 0.3% noble agar and vehicle control, or 100 μg/mL mouse immunoglobulin G (IgG) isotype, or anti-GPC1 mAb at various concentrations and placed on the base layer. After the upper layer of agar was solidified, 200 μL of complete culture medium containing the same control or mAb at the same concentration as the upper layer of agar was added to prevent desiccation. Colony formation by A549 and H460 cells was evaluated after 10–12 days of culture for number and size of colonies. Twenty-four hours prior to the evaluation, 200 μL of 1 mg/mL of MTT was added to each well for the evaluation of metabolic viability of the colonies [32]. Colonies were photographed using the Bio-Rad Gel Doc XR+ gel documentation system (Bio-Rad Laboratories, Inc. Hercules, CA, USA). The number and size of colonies were quantified using ImageJ (https://imagej.nih.gov/ij/). The effect of the antibody treatment was expressed by relative number of colonies [33] and percent of colony area [34].


$$Relative\;number\;of\;colonies\;\left(\text{\%}\right)=\frac{100\times {\left(Colony\;count\right)}_{treatment}}{{\left(Mean\;colony\;count\right)}_{vehicle\;control}}\;(1)$$

$$Colony\;area\;\left(\text{\%}\right)=\frac{100\times \left(Number\;of\;pixels\;in\;the\;region\;with\;an\;intensity\;above\;0\right)}{\left(Total\;number\;of\;pixels\;in\;the\;same\;region\right)}\;(2)$$

### 3-Dimentional spheroid invasion assay

To generate 3D tumor cell monoculture and tumor cell/fibroblast coculture spheroids, A549 or H460 cells alone (3,000 cells per well), or A549 or H460 cells mixed with LL97A fibroblasts at a ratio of 1:1 (a total of 3,000 cells per well), were added to a Spheron Nunclon 96-well plate (Thermo Fisher Scientific) followed by centrifugation at 400 × *g* for 10 min. After 3 days of incubation under the standard culture condition, spheroids were suspended in 1.5 mg/mL CultrexTM rat collagen I (R&D Systems; Minneapolis, MN, USA) diluted with the complete DMEM culture medium, and then transferred to a flat-bottom 96-well plate. After collagen solidified, complete DMEM culture medium was added to the top of the collagen matrix to provide a chemogradient for the spheroids. To evaluate the effect of anti-GPC1 mAb on cell invasion, anti-GPC1 mAb (50 μg/mL and 100 μg/mL) or mouse IgG isotype (100 μg/mL) was added directly to the collagen matrix to achieve the desired final concentration before solidification and to the complete culture medium on top of the solidified collagen matrix. Spheroids were maintained under the standard culture condition for 4 days to allow for invasion. Bright-field microscopy images were acquired daily on the EVOS^TM^ XL imaging system (Thermo Fisher Scientific, Waltham, MA, USA). Cell-covered area was measured using ImageJ (https://imagej.nih.gov/ij/).

### Transwell indirect co-culture system

The indirect tumor cell-fibroblast co-culture was carried out using a transwell system with Corning^TM^ Transwell^TM^ 24 mm inserts and 0.4 μm pore polyester membranes (Corning Inc., Corning, NY, USA). A549 and H460 lung tumor cells were seeded in the lower chamber at the density of 5 × 10E4 cells/well, while LL97A lung fibroblasts were in the upper chamber at the density of 10 × 10E4 cells/insert. Co-cultured cells were maintained in DMEM/F12 media containing 10% FBS in the absence and presence of vehicle, mouse IgG isotype control or anti-GPC1 mAb.

### Orthotopic A549-Red-Fluc lung tumor model and in vivo bioluminescence imaging

The orthotopic lung tumor model was established by intrapulmonary injection of A549-Red-Fluc cells in athymic nude mice using the method described elsewhere with slight modifications [35]. In brief, A549-Red-Fluc cells harvested during the exponential growth in vitro were suspended in PBS containing 10% Matrigel^®^ growth factor reduced (GFR) basement membrane matrix (Corning Inc., Corning, NY, USA) prior to the tumor cell inoculation procedure. Athymic nude mice were anesthetized under isoflurane and positioned in the lateral decubitus position with the left chest facing up and a small (about 5 mm) incision was made in the skin just below the scapula. Next, the chest wall soft tissue was gently spread to expose the thoracic ribs and intercostal space to allow visualization of the left lobe of the lung. At this stage, 50 μL of tumor cell suspension (1 × 10E6 A549-Red-Fluc cells in 50 μL PBS containing 10% Matrigel^®^ GFR) was drawn into a 3/10-cc insulin syringe equipped with a 31-G hypodermic needle (Becton, Dickinson and Company, Franklin Lakes, NJ, USA). Then the needle was inserted between the 6th and 7th ribs and the tumor cell suspension was injected 5 mm deep into the left lung parenchyma over a 1-minute period. After injection of the cells, the syringe was withdrawn from the tissue and the initial surgery incision made in the skin was closed with non-absorbable Ethilon^®^ 6-0 nylon sutures. After the orthotopic inoculation of tumor cells, weekly in vivo bioluminescence imaging (BLI) was performed throughout the experiment using the IVIS^®^ Spectrum in vivo imaging system (Perkin Elmer Inc., Waltham, MA, USA) to monitor changes in tumor burden over time in the same animals. To perform the in vivo BLI, tumor bearing mice received intraperitoneal (i.p.) injection of 150 mg/kg d-luciferin (Gold Biotechnology Inc., Olivette, MO, USA) 8 min before imaging and were anesthetized with isoflurane during imaging. Bioluminescence emitted from each tumor bearing animal was acquired using the Living Image Software (Perkin Elmer Inc., Waltham, MA, USA). Region of interest (ROI) measurements on the images were used to convert surface radiance to total flux of photons expressed in photons/second and used as readout for the orthotopic lung tumor size. For longitudinal comparison of bioluminescence, ROI was manually selected and kept consistent across all experiments.

### In vivo treatment protocol

Intravenous (i.v.) dosing regimen: to evaluate the effect of anti-GPC1 mAb on orthotopic lung tumor growth, athymic nude mice were randomly divided into (1) vehicle control, (2) mouse IgG isotype 10 mg/kg and (3) anti-GPC1 mAb 10 mg/kg groups once the orthotopic lung tumor lesions were detected by BLI in those animals, which was at 4–6 weeks after intrapulmonary tumor cell inoculation. Tumor-bearing animals were given i.v. injection of saline, 10 mg/kg of mouse IgG isotype and 10 mg/kg of anti-GPC1 mAb through tail vein once every 72 h for a total of 8 doses. All animals were euthanized 24 h after the last dose. Plasma, lung tissue and tumor tissue samples collected from individual animals were stored at −80℃ before subjected to further analyses.

I.p. dosing regimen: to evaluate the efficacy of anti-GPC1 mAb treatment on orthotopic lung tumor growth, athymic nude mice were randomly divided into four groups: (1) vehicle control, (2) anti-GPC1 mAb 1 mg/kg, (3) anti-GPC1 mAb 10 mg/kg and anti-GPC1 mAb 50 mg/kg groups. Two weeks after the orthotopic tumor cell inoculation, individual animals received i.p. injection of saline or anti-GPC1 mAb at different dose levels once a week for 3 doses (i.e., on Day 0, 7 and 14) and then once every 10 days for 2 more doses (i.e., on Day 24 and 34). Animals were euthanized 1 week after the last dose (i.e., on Day 41). Superficial and internal macroscopic tumors in the lungs were assessed at necropsy. Those that failed to develop lung tumors were excluded from further analyses. Plasma, lung tissue and tumor tissue samples collected from individual tumor-bearing animals were stored at −80℃ before subjected to further analyses.

The method of euthanasia for athymic nude mice used in this study was exsanguination under isoflurane anesthesia and the death was confirmed by cervical dislocation of the neck prior to tumor tissue resection.

### Determination of anti-GPC1 mAb levels in plasma using enzyme-linked immunosorbent assay

A 96-well Immulon^®^ Microtiter^TM^ plate (Thermo Fisher Scientific, Waltham, MA, USA) was coated with 50 μL of recombinant human GPC1 protein (2.5 μg/mL in PBS) for 1 h at 37℃. After the coated plate was washed twice with PBS containing 0.1% Tween 20 (PBST) and blocked with 100 μL/well of 1.5% BSA in PBST at 37℃ for 30 min, an aliquot of 100 μL of diluted plasma sample (1:200 dilution in PBST containing 1.5% BSA) was added to each well and incubated at 37℃ for 1 h. After the plasma samples were removed, the plate was washed four times with PBST and then incubated with 100 μL/well of diluted goat anti-mouse-horseradish peroxidase (HRP) antibody (1:4,000 dilution in PBST containing 1.5% BSA; Southern Biotech, Birmingham, AL, USA) at 37℃ for 1 h. For colorimetric enzyme-linked immunosorbent assay (ELISA) detection, 100 μL of 3,3’,5,5’-tetramethylbenzidine (TMB; Surmodics IVD, Inc., Eden Prairie, MN) was added to each well. After the plate was incubated at room temperature for 10–15 min, the enzymatic reaction was stopped by adding 100 μL of 0.4 mol/L of H_2_SO_4_ to each well. The plate was read at 450 nm on a SpectraMax 190 microplate reader equipped with SoftMax Pro software (Molecular Devices, Sunnyvale, CA, USA). The optical density was used to estimate plasma levels of anti-GPC1 mAb.

### Western blot analysis

Monocultured and co-cultured A549, H460 and LL97A cells, and normal lung and A549 lung tumor tissue samples collected from the in vivo studies were subjected to semi-quantitative Western blot analysis to determine the phosphorylation of downstream effectors of FGFR signaling, including the MEK/ERK/RSK, Akt/GSK3/mTOR and Src/focal adhesion kinase (FAK) pathways, as well as the protein expression of GPC1 and selected epithelial-mesenchymal transition (EMT) markers. A complete list of primary antibodies used in the Western blot analysis is shown in Table S1.

### Statistical analysis

Statistical analyses were performed using Number Cruncher Statistical Systems 2007 (Keysville, UT, USA). Data are presented as the mean ± standard deviation (SD) unless otherwise indicated. The two-sample *t*-test was used to determine if there was a statistically significant difference between the means of two independent groups. Comparison of means between more than two independent groups was made using the one-way ANOVA followed by Tukey-Kramer post hoc multiple comparison test. To compare the tumor growth rate among three study groups, tumor sizes were transformed with square root and linear regressions were applied to fit the tumor growth curves for individual mice. When three or more independent sample data did not follow a normal distribution, a nonparametric Kruskal-Wallis test was used to compare the independent groups instead of one-way ANOVA. Pearson’s correlation coefficient was used to describe the strength of linear correlation between two variables. A two-sided *P*-value of less than 0.05 was considered statistically significant.

## Results

### In vitro anti-GPC1 mAb treatment exhibited greater cytotoxic potential against LL97A lung fibroblasts, A549/LL97A and H460/LL97A coculture spheroids than against A549 and H460 monocultures

**Figure 1:**
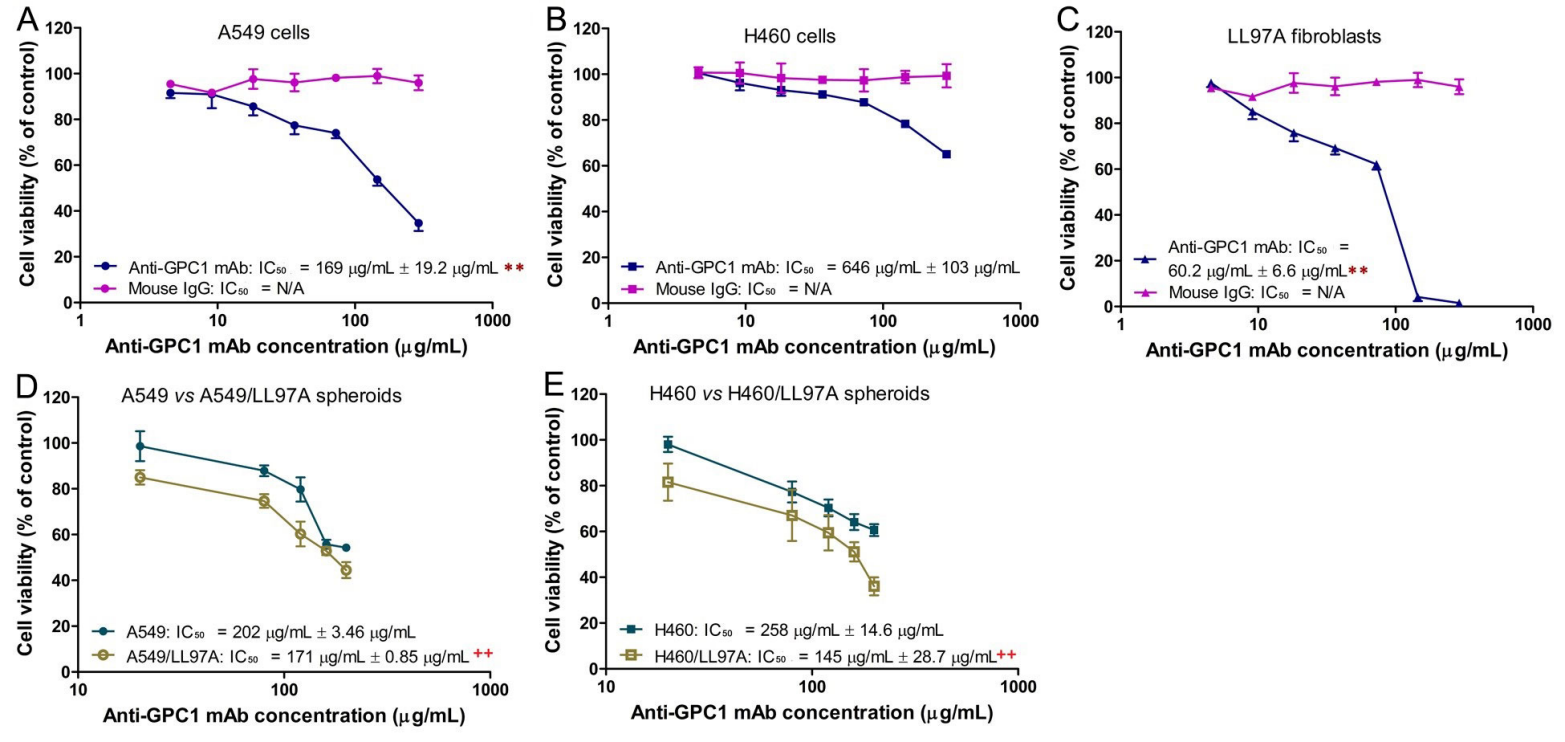
Determination of cytotoxicity of anti-GPC1 mAb in 2D monocultures and 3D monoculture and coculture spheroids. (A) 2D A549 monocultures; (B) 2D H460 monocultures; (C) 2D LL97A monocultures; (D) 3D A549 monocultures and A549/LL97A cocultures; (E) H460 monocultures and H460/LL97A cocultures. Cells were treated with anti-GPC1 mAb and mouse IgG isotype at a range of concentration. IC50 values were based on the concentration-effect curves. Results are presented as mean ± SD (*n* = 4 for 2D cultures and *n* = 3 for 3D cultures). Error bars represent the inter-assay SD. **P < 0.01 compared with anti-GPC1 mAb treated 2D H460 monocultures using one-way ANOVA followed by Tukey-Kramer post hoc multiple comparison test. ++*P* < 0.01 compared with the corresponding 3D monoculture counterpart using independent two-sample *t*-test. GPC1: glypican-1; mAb: monoclonal antibody; IgG: immunoglobulin G

Evaluation of the in vitro cytotoxic effect of anti-GPC1 mAb in 2D A549, H460 and LL97A monocultures was carried out using the MTT assay after the cells were treated with anti-GPC1 mAb or mouse IgG isotype at concentrations ranging from 4.5 μg/mL to 290 μg/mL for 72 h. The effect of anti-GPC1 mAb on the viability of 3D A549 and H460 monoculture spheroids and A549/LL97A and H460/LL97A coculture spheroids was determined using the CellTiter-Glo 3D reagent that generates the stable ATP-dependent luminescent signal. Figure 1 shows the concentration-effect curves for anti-GPC1 mAb and mouse IgG isotype treatments in different cell lines. IC_50_ values were calculated by fitting the log(inhibitor) vs. normalized response model to the data using non-linear regression in GraphPad Prism 8.0.1 (GraphPad Software. Boston, MA, USA). The difference in the IC_50_ values was then compared between the two treatment groups in individual cell lines. Results of the MTT assay showed that the IC_50_ values of anti-GPC1 mAb for A549 NSCLC cells and LL97A lung fibroblasts were significantly lower than that for H460 cells (*P* < 0.01 for both; Figure 1A–C). Among the three cell lines tested, LL97A lung fibroblasts were shown with the lowest IC_50_ value, suggesting that the lung fibroblasts are more sensitive to anti-GPC1 mAb treatment than the NSCLC cells. Treatment with the mouse IgG isotype for 72 h had no effect on the viability of all three cell lines tested (Figure 1A–C). Results of the 3D cell viability assay indicated that the IC_50_ values of anti-GPC1 mAb in A549/LL97A and H460/LL97A coculture spheroids were 15% and 44% lower than those in A549 and H460 monoculture spheroids, respectively, (*P* < 0.01 for both; Figure 1D–E), suggesting tumor cell-fibroblast cocultures are more sensitive to the cytotoxic effect of anti-GPC1 mAb than tumor cell monocultures.

### Anti-GPC1 effectively inhibited anchorage-independent growth of A549 and H460 tumor cells

**Figure 2:**
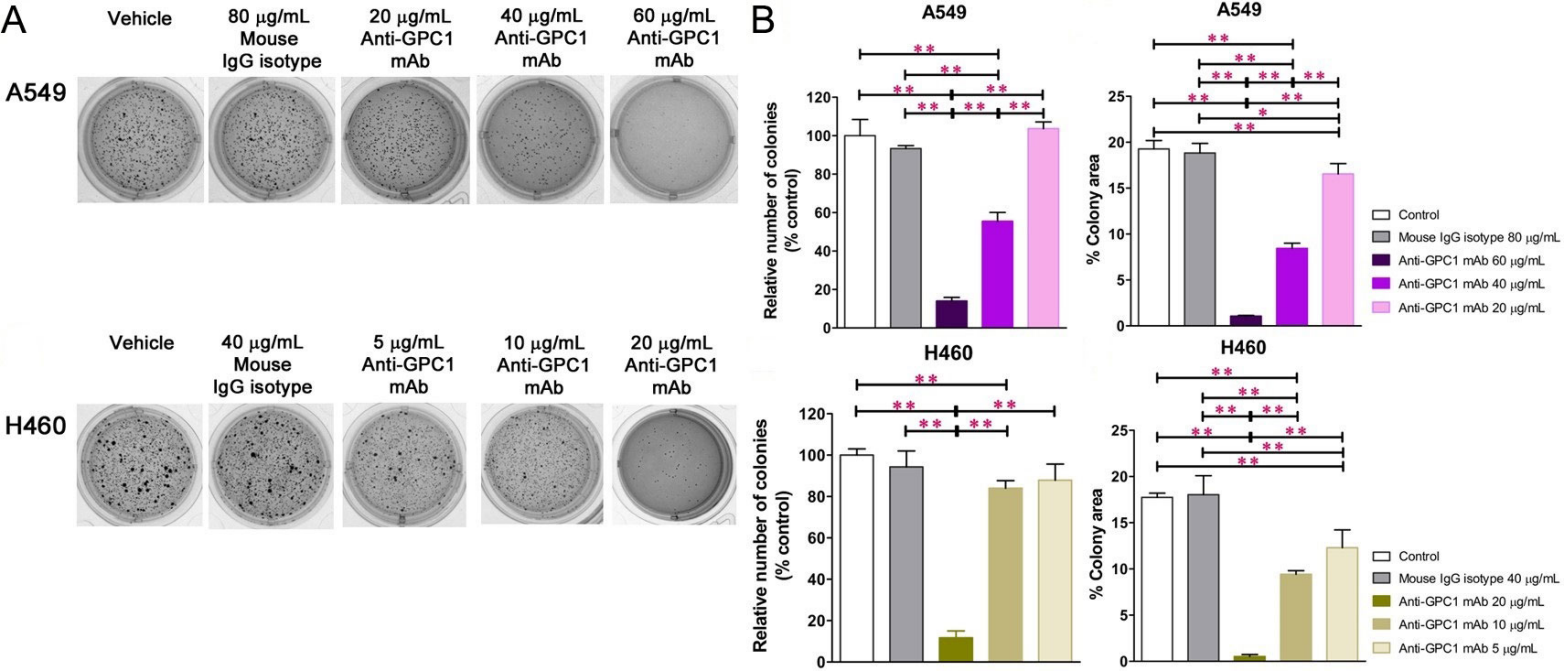
Evaluation of the inhibitory effect of anti-GPC1 mAb on tumor cell colony formation in soft agar cultures. Soft agar colony formation assay for assessing the effect of anti-GPC1 mAb on the anchorage-independent growth of A549 and H460 NSCLC cells after treatment with increasing concentrations of anti-GPC1 mAb. Colonies > 0.1 mm were counted using ImageJ software. Independent experiments were performed in quadruplicates (*n* = 4) with 2 × 10E3 cells/well plated in a 24-well plate for the assay. Colonies were analyzed after 10–12 days of culture and stained with 1 mg/mL of MTT. (A) Representative images of colony formation of each cell line from one experiment are presented; (B) quantitative analyses of colony formation and anchorage-independent growth based on the relative number of colonies and percent of colony area, respectively. Results were presented as the mean ± SD (*n* = 4). SD is denoted by the error bars. *P < 0.05 and **P < 0.01 compared among vehicle, mouse IgG isotype and individual anti-GPC1 mAb treatment groups in the same cell line using one-way ANOVA followed by Tukey-Kramer post hoc multiple comparison test. GPC1: glypican-1; mAb: monoclonal antibody; IgG: immunoglobulin G

In soft agar colony formation assay, tumor cells were deprived of their anchorage by being suspended in 0.3% noble agar as single cells (Figure 2A). The relative number of colonies [colony area (%)] and percent of well area covered by colonies [colony area (%)] were determined to quantify the inhibitory effect of anti-GPC1 mAb on tumor cell survival and proliferation under the anchorage-independent condition. For A549 cells, treatment with 40 μg/mL and 60 μg/mL of anti-GPC1 mAb resulted in a significantly less number of colonies as compared with the number of colonies formed in the vehicle control and 80 μg/mL mouse IgG isotype groups (*P* < 0.01 for all; Figure 2B). Treatment with 20 μg/mL, 40 μg/mL and 60 μg/mL of anti-GPC1 mAb also significantly inhibited the anchorage-independent growth of A549 cells, which was reflected by the significant decrease in the percent colony areas in the anti-GPC1 mAb treated A549 cells as compared with those in A549 cells treated with the vehicle control or 80 μg/mL mouse IgG isotype. For H460 cells, treatment with 10 μg/mL and 20 μg/mL of anti-GPC1 mAb resulted in a significant decrease in the relative number of colonies formed as compared with the vehicle treatment (*P* < 0.01 for all; Figure 2B). Treatment with 5 μg/mL, 10 μg/mL and 20 μg/mL of anti-GPC1 mAb significantly decreased the colony areas (%) as compared with treatment with vehicle or 40 μg/mL of mouse IgG isotype. The inhibitory effect of anti-GPC1 mAb on the colony formation was concentration dependent irrespective of the cell line used (*P* < 0.01 for all; Pearson’s correlation). It was noted that the estimated concentrations at which 50% of colony formation was inhibited were significantly lower than the IC_50_ values obtained from the MTT assay performed in 2D cell culture (41.2 μg/mL ± 0.93 μg/mL vs. 169 μg/mL ± 19.2 μg/mL for A549 cells; 13.6 μg/mL ± 0.59 μg/mL vs. 646 μg/mL ± 103 μg/mL for H460 cells. *P* < 0.01 for both; Figure 1A, 1B and 2B). This finding suggests that the anchorage-independent tumor cell growth is more sensitive to anti-GPC1 mAb treatment.

### Anti-GPC1 mAb reduced the invasion capacity of tumor cell/fibroblast coculture spheroids

**Figure 3:**
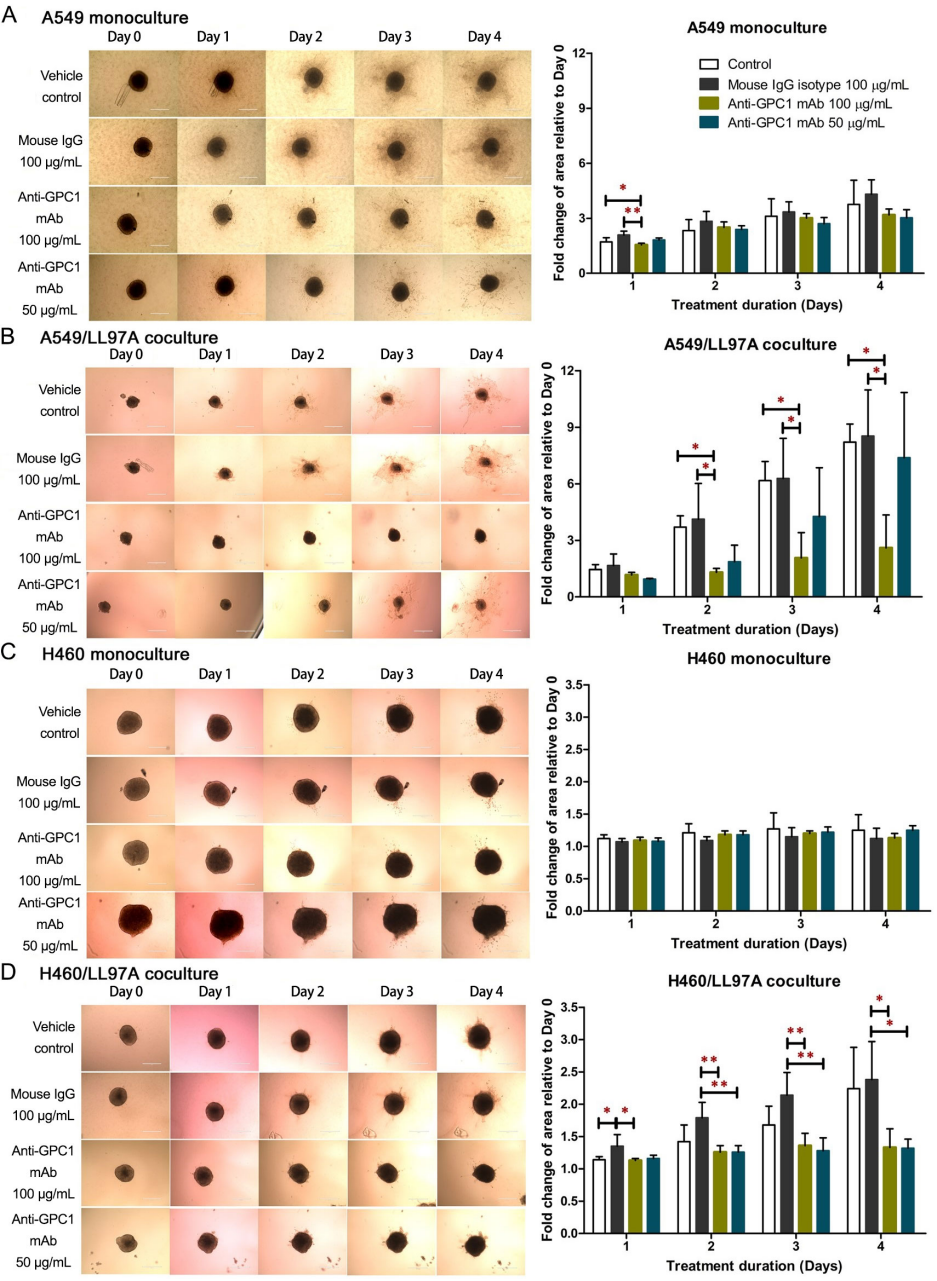
Evaluation of the anti-invasive effect of anti-GPC1 mAb using the 3D spheroid invasion assay. (A) A549 monoculture spheroids, (B) H460 monoculture spheroids, (C) A549 coculture spheroids; (D) H460 coculture spheroids. Cell-covered area was quantified using ImageJ and expressed as the fold change in area relative to Day 0. Daily micrographs showed the invasion progress of representative spheroids. Original magnifications, × 100. Error bars represent the SD of the mean from eight independent experiments (*n* = 4). Results are presented as mean ± SD. *P < 0.05 and **P < 0.05 compared among vehicle, mouse IgG isotype and individual anti-GPC1 mAb treatment groups using one-way ANOVA followed by Tukey-Kramer post hoc multiple comparison test. GPC1: glypican-1; mAb: monoclonal antibody; IgG: immunoglobulin G

The 3D spheroid invasion assay was conducted to evaluate the effect of anti-GPC1 mAb on the invasion potential of A549 and H460 tumor cells that were either monocultured or cocultured with LL97A fibroblasts (Figure 3). The final concentrations of anti-GPC1 mAb used in monoculture and coculture spheroids were 50 μg/mL and 100 μg/mL. As indicated by the fold change in cell-covered area relative to Day 0, treatment with anti-GPC1 mAb at 50 μg/mL and 100 μg/mL had little effect on the invasion potential of A549 and H460 monoculture spheroids as compared with the vehicle control or 100 μg/mL mouse IgG isotype treatment (Figure 3A and 3C). GPC1 is known to function as a co-receptor/modulator of various growth factors to enhance the binding to their respective receptors [4–11], and cancer-associated fibroblasts (CAFs) are an important contributor of some of those growth factors in tumors, including FGF2 and TGFβ [36, 37]. In this regard, the tumor cell/fibroblast coculture spheroids were used to evaluate the anti-invasion potential of anti-GPC1 mAb. A549/LL97A coculture spheroids treated with 100 μg/mL of anti-GPC1 mAb exhibited significantly reduced cell-covered areas compared with those treated with vehicle control or 100 μg/mL of mouse IgG isotype from Day 2 through 4 (*P* < 0.05 for all; Figure 3B). In H460/LL97A coculture spheroids, treatment with anti-GPC1 mAb at 50 and 100 μg/mL resulted in significantly less cell-covered areas than treatment with 100 μg/mL of mouse IgG isotype on Day 2 (*P* < 0.01 for both), Day 3 (*P* < 0.01 for both) and Day 4 (*P* < 0.05 for both; Figure 3D). Overall, these results suggest that anti-GPC1 mAb treatment inhibits fibroblast-led collective tumor cell invasion.

### Evaluation of molecular changes in response to anti-GPC1 mAb treatment in monocultured and co-cultured NSCLC cells and lung fibroblasts

**Figure 4:**
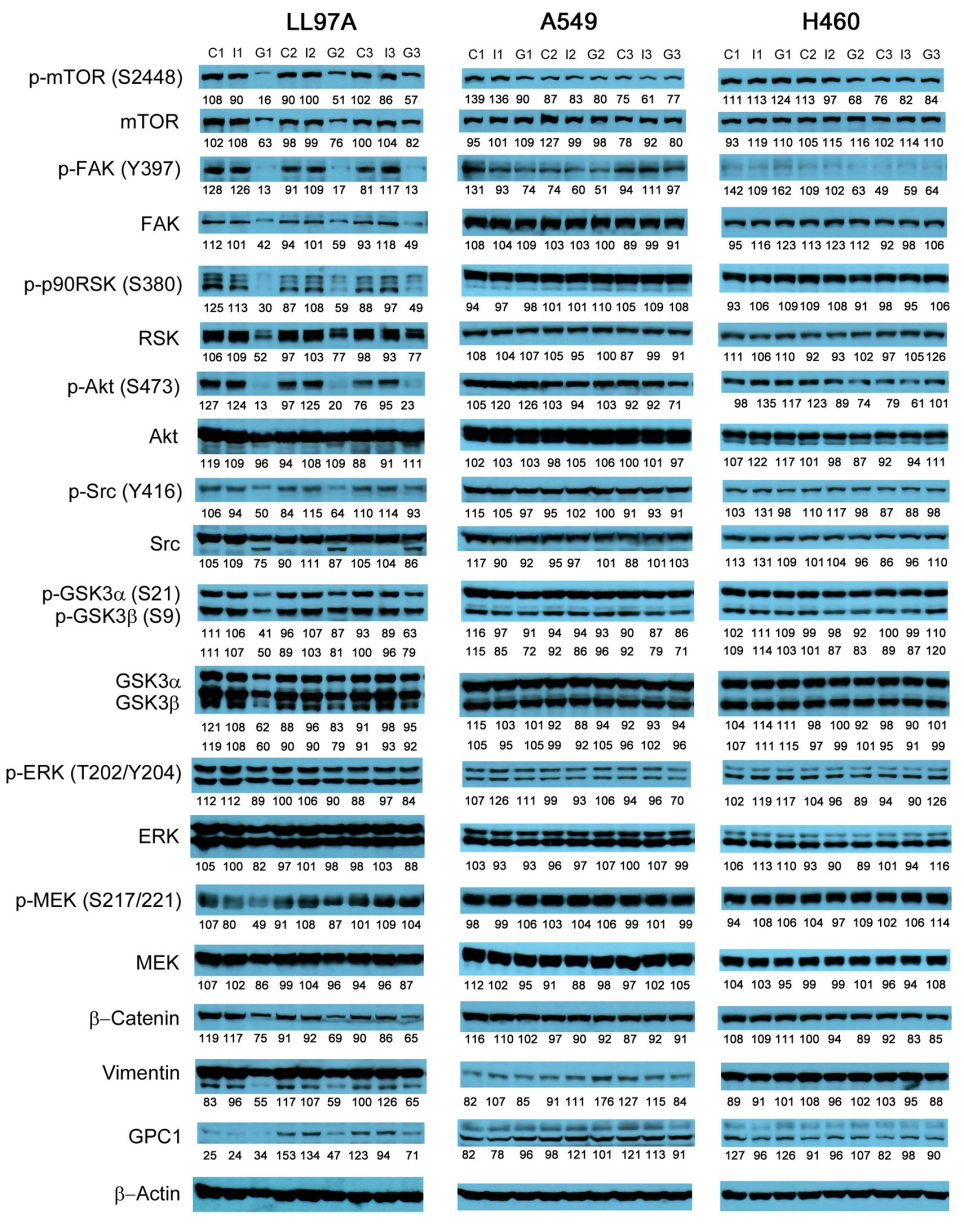
Western blot analysis of monocultured LL97A, A549 and H460 cells after treatments. Western blot images demonstrated the protein expression of total and phosphorylated mTOR, RSK1/2/3, FAK, Src Akt, GSK3α/3β, ERK1/2 and MEK1/2, selected EMT markers and GPC1 protein in monocultured LL97A lung fibroblasts, A549 and H460 tumor cells treated with vehicle control, 100 μg/mL mouse IgG isotype, or 100 μg/mL anti-GPC1 mAb for 24 h. Numbers below individual blots represent the percentage of the normalized integrated density values against β-actin (loading control). C: control; I: mouse IgG; G: anti-GPC1 mAb. GPC1: glypican-1; p-FAK: phospho-focal adhesion kinase; p90RSK: 90 kDa ribosomal s6 kinase; GSK3α: glycogen synthase kinases 3α; ERK: extracellular signal-regulated kinase; MEK: mitogen-activated protein kinase kinase; GPC1: glypican-1

**Figure 5:**
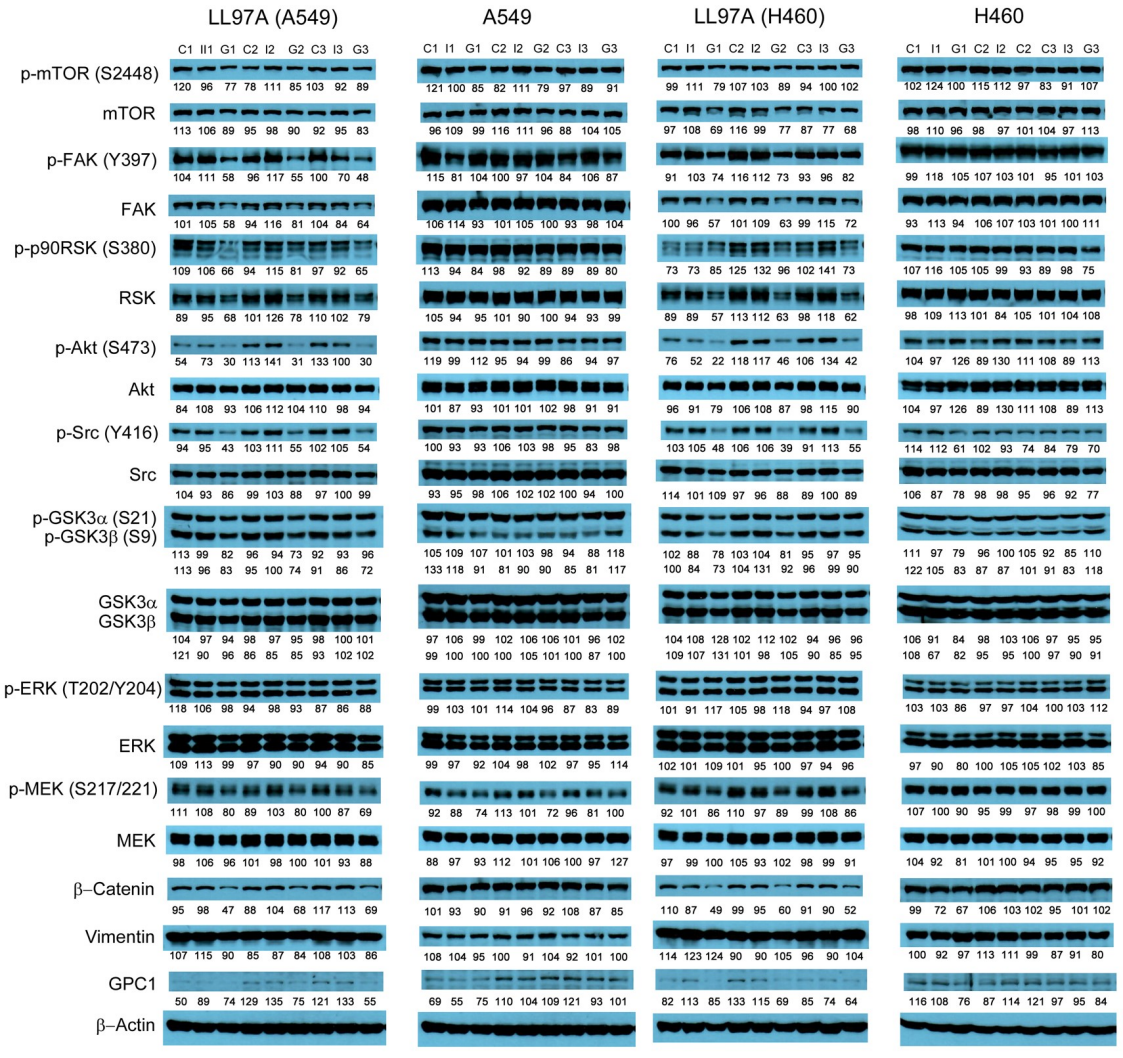
Western blot analysis of co-cultured LL97A, A549 and H460 cells after treatments. Western blot images demonstrated the expression of total and phosphorylated mTOR, RSK1/2/3, FAK, Src Akt, GSK3α/3β, ERK1/2 and MEK1/2, selected EMT markers and GPC1 protein in LL97A lung fibroblasts, A549 and H460 tumor cells that were co-cultured in a transwell indirect co-culture system and treated with vehicle control, 100 μg/mL mouse IgG isotype, or 100 μg/mL anti-GPC1 mAb for 24 h. Numbers below individual blots represent the percentage of the normalized integrated density values against β-actin (loading control). C: vehicle control; I: 100 μg/mL mouse IgG isotype; G: 100 μg/mL anti-GPC1 mAb. p-FAK: phospho-focal adhesion kinase; p90RSK: 90 kDa ribosomal s6 kinase; GSK3α: glycogen synthase kinases 3α; ERK: extracellular signal-regulated kinase; MEK: mitogen-activated protein kinase kinase; GPC1: glypican-1

**Figure 6:**
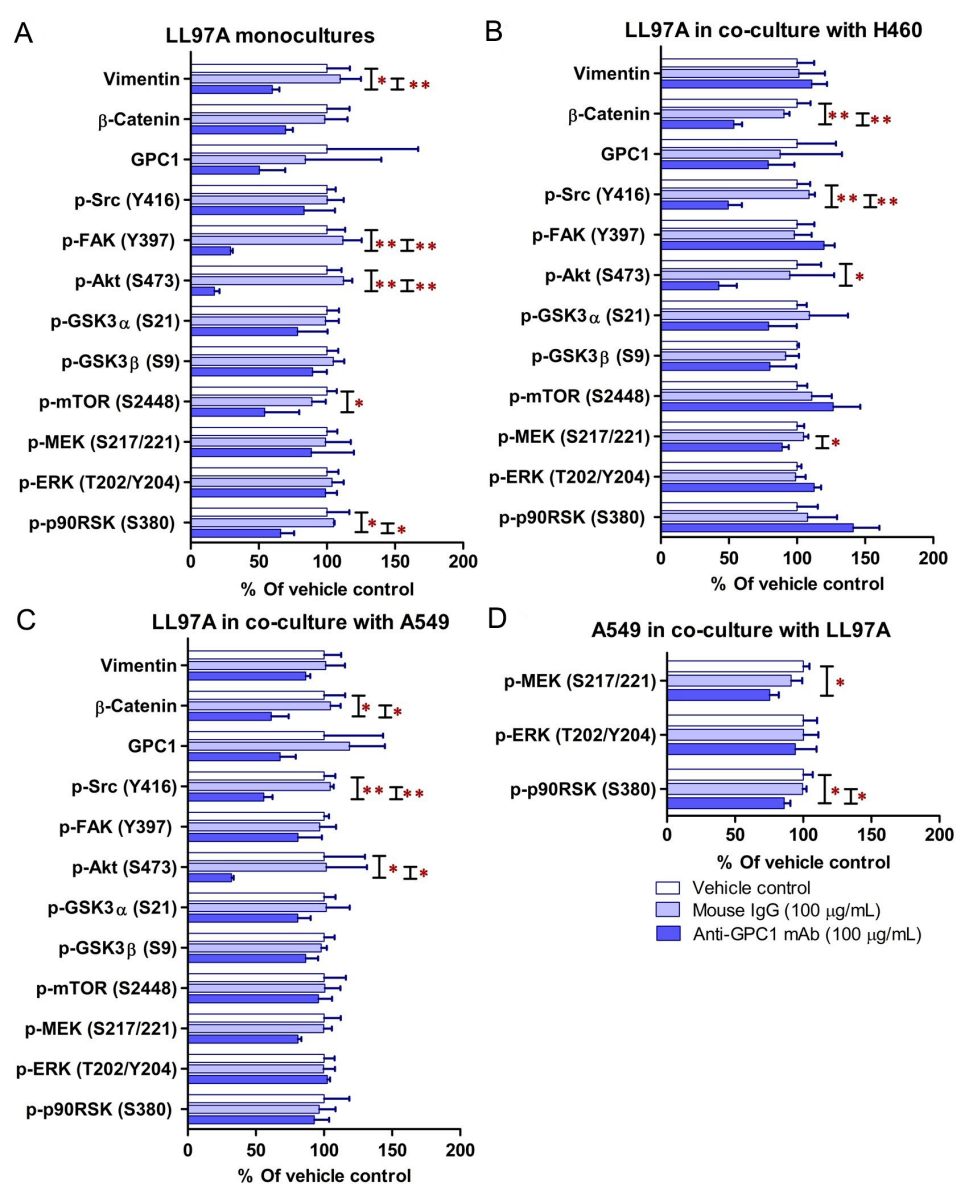
Semi-quantitation of protein expression in monoculture and coculture samples determined by Western blot analysis. (A) LL97A monocultures; (B) LL97A fibroblasts in indirect coculture with H460 cells; (C) LL97A fibroblasts in indirect coculture with A549 cells; (D) A549 cells in indirect coculture with LL97A fibroblasts. Effects of anti-GPC1 mAb on the expression levels of selected EMT markers and effector proteins common to the GPC1-facilitated growth factor signal transduction pathways, including MEK/ERK/RSK, Akt/GSK3/mTOR, Src/FAK pathways were determined using Western blot analysis. Cell monocultures or co-cultures were treated with vehicle (i.e., PBS), 100 μg/mL mouse IgG isotype and 100 μg/mL anti-GPC1-mAb for 24 h. Whole cell lysate samples were subjected to Western blot analysis (30 μg of protein per lane). Detection of β-actin was used to ensure equal sample loading per lane. Relative immunoreactive band intensities are expressed as percent change over the average signal value in the vehicle-treated cells. For phosphorylated proteins, results are expressed as the ratio of phosphorylated-to-total species relative to the vehicle control cells. Data are representative of three independent experiments (*n* = 3) and presented as mean ± SD. SD is denoted by the error bars. *P < 0.05 and **P < 0.01 compared among vehicle, 100 μg/mL mouse IgG isotype and 100 μg/mL anti-GPC1-mAb in the same cell line using one-way ANOVA followed by Tukey-Kramer post hoc multiple comparison test. GPC1: glypican-1; p-FAK: phospho-focal adhesion kinase; GSK3α: glycogen synthase kinases 3α; MEK: mitogen-activated protein kinase kinase; ERK: extracellular signal-regulated kinase; p90RSK: 90 kDa ribosomal s6 kinase

Since GPC1 is believed to facilitate the activation of signaling pathways downstream of the receptors of HB growth factor family of proteins, it is anticipated that anti-GPC1 mAb blocks the interaction between GPC1 and those growth factors, and subsequently hinders the activation of the cognate receptors and downstream pathways. In this study, A549, H460 and LL97A cells that were cultured as monocultures or co-cultured in an indirect transwell coculture system were exposed to vehicle control, 100 μg/mL of mouse IgG isotype or 100 μg/mL of anti-GPC1 mAb for 24 h. The collected whole cell lysate samples were subjected to Western blotting analysis to examine the effect of in vitro anti-GPC1 mAb treatment on common downstream growth factor receptor signaling pathways, including the MEK/ERK/RSK, Akt/GSK3/mTOR and Src/FAK pathways, as well as the protein expression of GPC1 and selected EMT markers, including β-catenin and vimentin. As shown in Figures 4 and 5, GPC1 protein expression was detected in all three cell lines by Western blotting, indicating that the target antigen of anti-GPC1 mAb was present in all three cell lines tested. In monocultures, treatment with anti-GPC1 mAb had no effect on the levels of phosphorylated signaling effectors and the protein expression of GPC1, β-catenin and vimentin proteins in A549 and H460 NSCLC cell lines (Figure 4). However, in LL97A lung fibroblast monocultures, as compared with both of the vehicle control and mouse IgG isotype control treatment, anti-GPC1 mAb treatment significantly decreased the FAK phosphorylation at Tyr397 (*P* < 0.01 for both), RSK phosphorylation at Ser380 (*P* < 0.05 for both), Akt phosphorylation at Ser473 (*P* < 0.01 for both), and vimentin protein expression (*P* < 0.05 compared with the vehicle control and *P* < 0.01 compared with the mouse IgG isotype; Figure 6A). Moreover, compared with the vehicle control treatment, the anti-GPC1 mAb treatment significantly decreased the expression of phospho-mTOR at Ser2448 in LL97A fibroblasts (*P* < 0.05; Figure 6A). These data indicated that the anti-GPC1 mAb treatment significantly decreased the activities of FAK, Akt, mTOR and RSK in LL97A lung fibroblasts, suggesting that the antiproliferative effect of anti-GPC1 mAb on LL97A lung fibroblasts is attributable to its inhibitory effect on the FAK/Src, Akt/mTOR, MEK/ERK/RSK pathways (Figure 1C and Figure 6A). The significant decrease in vimentin protein expression in anti-GPC1 treated LL97A cells may be associated with the suppression of fibroblasts proliferation and activity in vitro (Figure 6A). When LL97A lung fibroblasts were co-cultured indirectly with H460 lung tumor cells, anti-GPC1 mAb treatment significantly decreased the phosphorylation of Src at Tyr416 and β-catenin protein expression as compared with both of the vehicle control and mouse IgG isotype control treatment (*P* < 0.01 for all; Figure 5 and Figure 6B). Moreover, the expression of phospho-Akt at Ser473 in anti-GPC1 mAb treated LL97A fibroblasts was significantly lower than that in the vehicle control-treated LL97A fibroblasts (*P* < 0.05), while the expression of phospho-MEK at Ser217/221 in anti-GPC1 mAb treated LL97A fibroblasts was significantly lower than that in the mouse IgG isotype-treated LL97A fibroblasts (*P* < 0.05; Figure 6B). When LL97A lung fibroblasts were co-cultured indirectly with A549 cells, the anti-GPC1 mAb treatment significantly decreased the expression of phospho-Src (Tyr416; *P* < 0.01 for both), phospho-Akt (Ser473; *P* < 0.05 for both) and β-catenin protein (*P* < 0.01 for both) as compared with both of the vehicle control and mouse IgG isotype treatment (Figure 5 and Figure 6C). In addition, in the co-cultured A549 cells, the anti-GPC1 mAb treatment significantly decreased the RSK phosphorylation at Ser380 (*P* < 0.05 compared with both of the vehicle and mouse IgG isotype controls) and MEK phosphorylation at Ser217/221 (*P* < 0.05 compared with the vehicle control; Figure 6D). Taken together, results of the Western blot analysis of the in vitro monoculture and co-culture samples suggest that lung fibroblasts are more sensitive to anti-GPC1 mAb than lung tumor cells and the interaction between NSCLC cells and lung fibroblasts influences the molecular response to anti-GPC1 mAb treatment.

### In vivo response of orthotopic A549 xenograft models to anti-GPC1 mAb treatment

**Figure 7:**
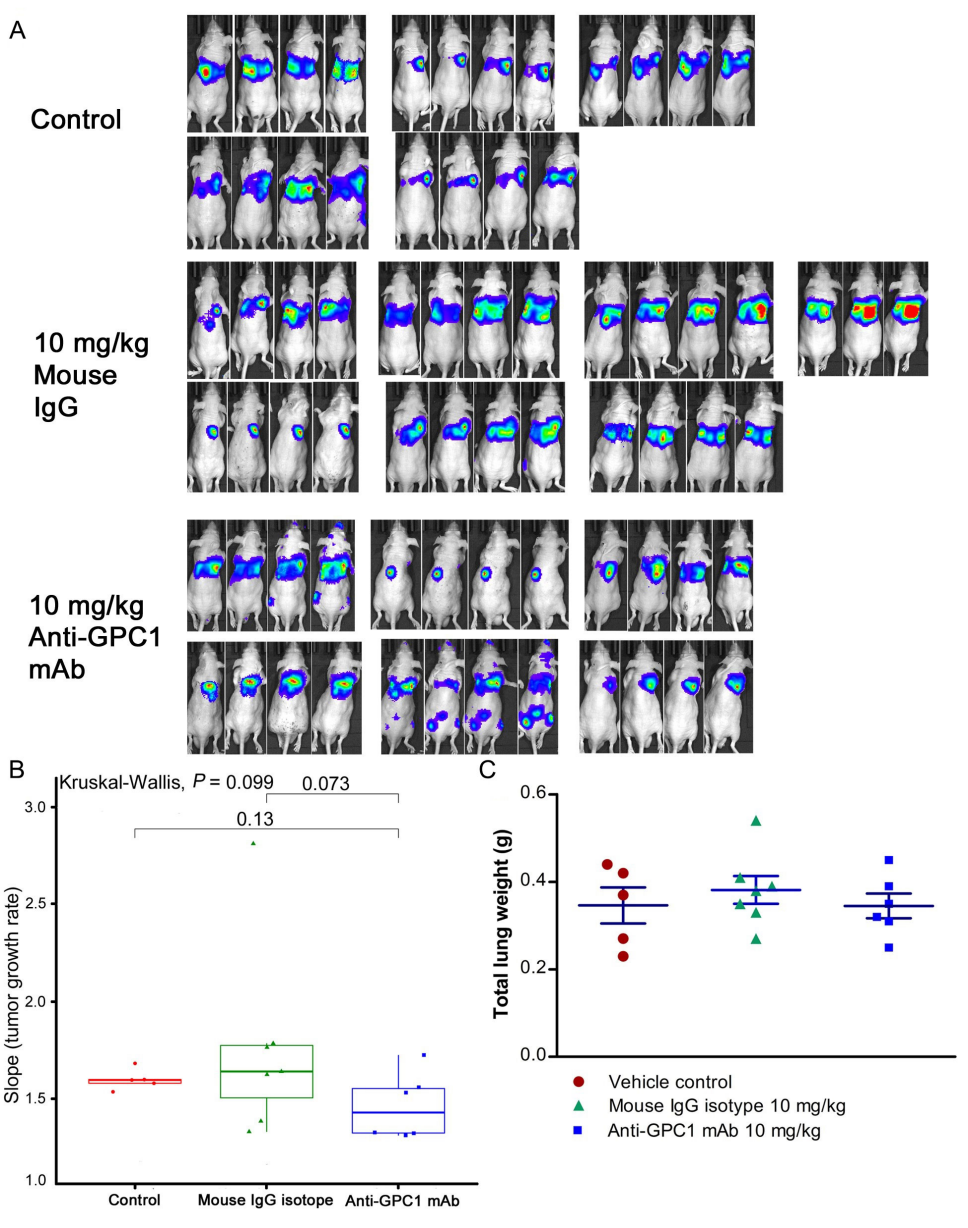
Assessment of the growth of established orthotopic A549 lung tumors in response to anti-GPC1 mAb treatment. (A) Assessment of orthotopic A549 lung tumor growth using BLI. BLI was conducted once a week over the 24 days of treatment period during which the tumor bearing athymic nude mice were given tail-vein injections of vehicle control (*n* = 5), or 10 mg/kg mouse IgG isotype (*n* = 7), or 10 mg/kg of anti-GPC1 mAb (*n* = 7) once every 72 h for a total of 8 doses; (B) comparison of tumor growth rates among study groups. Tumor growth rate was determined from serial measurements of tumor volume obtained from BLI photon counts. A nonparametric Kruskal-Wallis test was used to compare the tumor growth rate among control, mouse IgG isotype and anti-GPC1 mAb groups, and no statistically significant difference was found among the three study groups (*P* > 0.05); (C) comparison of total lung weights among study groups. No significant difference in total lung weights that reflect the lung tumor burden was found among three study groups using one-way ANOVA followed by Tukey-Kramer post hoc multiple comparison test (*P* > 0.05). Data are presented as mean ± SD. SD is denoted by the error bars. IgG: immunoglobulin G; GPC1: glypican-1; mAb: monoclonal antibody

**Figure 8:**
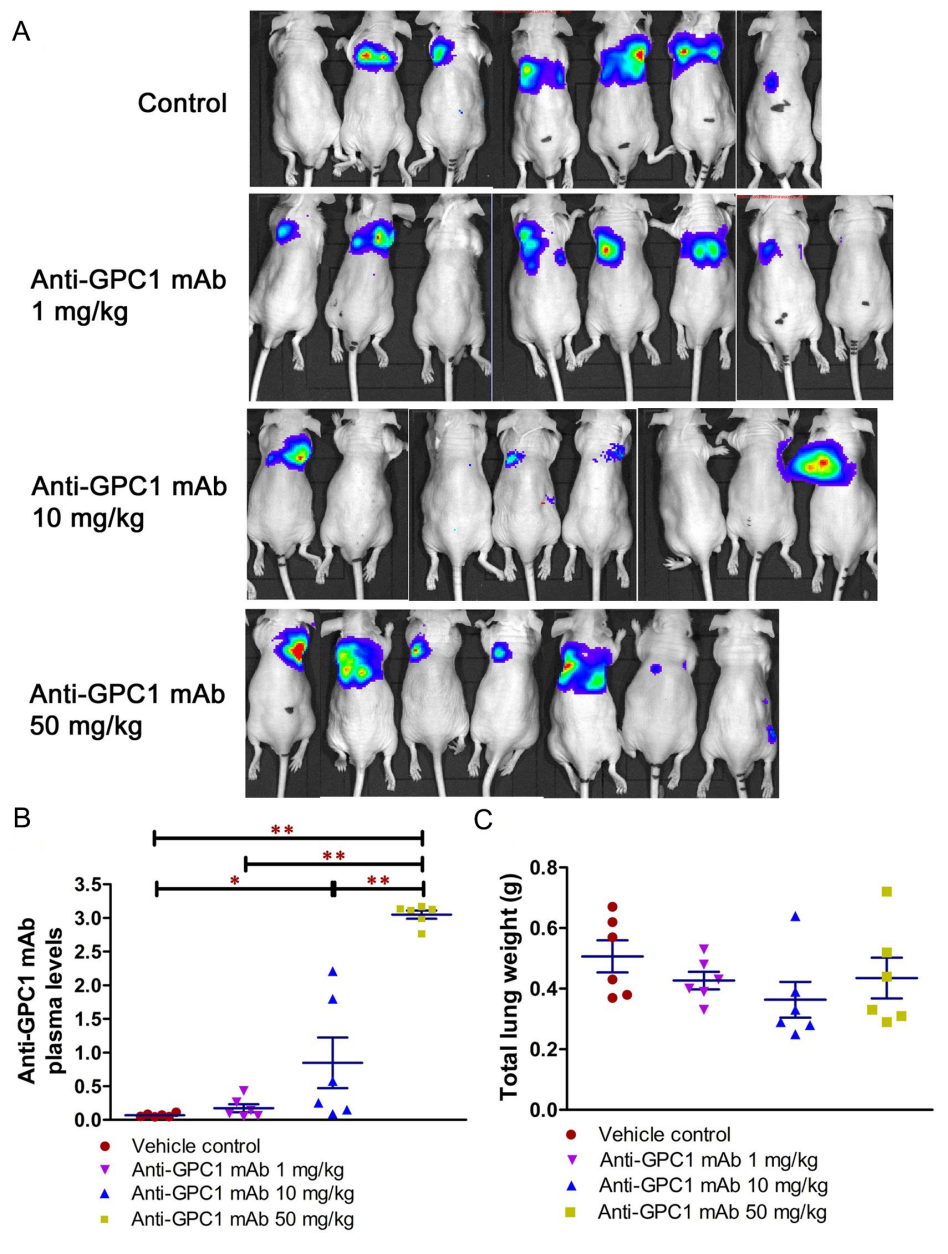
Evaluation of the anti-tumor of anti-GPC1 mAb in an orthotopic A549 lung tumor model following the intraperitoneal (i.p.) administration of the mAb. (A) Bioluminescence imaging of orthotopic A549 lung tumor performed a week after the last dose; (B) anti-GPC1 mAb plasma levels on the final day of the experiment; (C) weight measurement of lungs collected from mice bearing orthotopic A549 lung tumors. Orthotopic A549 lung tumor growth in response to i.p. administration of anti-GPC1 mAb at the dose level of 1, 10 and 50 mg/kg was assessed using BLI and total lung weight. Two weeks after orthotopic inoculation of A549 tumor cells, athymic nude mice received i.p. injections of PBS (vehicle) or anti-GPC1 mAb at 1, 10 and 50 mg/kg once a week for 3 doses and then once every 10 days for 2 more doses. Bioluminescence signals were detected in 6 out of 7 (6/7), 6/8, 4/8, and 6/7 animals in the vehicle control, 1 mg/kg, 10 mg/kg and 50 mg/kg anti-GPC1 mAb groups, respectively. Anti-GPC1 mAb plasma levels determined by ELISA showed a dose-dependent increase in the anti-GPC1 mAb plasma levels. No significant difference in total lung weights that reflect the lung tumor burden was found among the four study groups *P* > 0.05. Data are presented as mean ± SD (*n* = 6 for each study group). SD is denoted by the error bars. *P < 0.05 and **P < 0.01 compared among vehicle control, 1 mg/kg, 10 mg/kg and 50 mg/kg anti-GPC1 mAb groups using one-way ANOVA followed by Tukey-Kramer post hoc multiple comparison test. GPC1: glypican-1; mAb: monoclonal antibody

Given the potential impact of anti-GPC1 mAb on the interaction between tumor cells and tumor associated fibroblasts observed in the in vitro model, the anti-tumor activity of anti-GPC1 mAb was evaluated and associated mechanisms were explored using an orthotopic A549 tumor xenograft model. In the initial study, the athymic nude mice bearing established orthotopic A549 lung tumors were given tail-vein injections of vehicle (saline) control, or 10 mg/kg mouse IgG isotype, or 10 mg/kg of anti-GPC1 mAb once every 72 h for a total of 8 doses. The treatment was started once the progression of orthotopic lung tumor growth was confirmed by the in vivo BLI, which was 4–6 weeks after the intrapulmonary inoculation of luciferase expressing A549 tumor cells. BLI was conducted once a week over the 24 days of treatment period (Figure 7A). The relative rate of lung tumor growth in the anti-GPC1 mAb group, which was calculated as the slope of the curve on the semi-logarithmic plot of BLI total signal intensity over time, was lower than those in the vehicle control and mouse IgG isotype groups. Since the lung tumor growth rate data did not follow a normal distribution, a nonparametric Kruskal-Wallis test was used to test the difference between study groups. The result showed that the difference was not statistically significant (*P* > 0.05; Figure 7B). Likewise, the difference in total lung weights that reflect the lung tumor burden was not statistically significant among the three study groups (*P* > 0.05; Figure 7C). In the second study, athymic nude mice were given the i.p. injections of anti-GPC1 mAb at 1 mg/kg, 10 mg/kg and 50 mg/kg once a week for 3 doses and then once every 10 days for 2 more doses. The treatment was initiated 2 weeks after the orthotopic inoculation of A549 cells for early blockage of GPC1 activity. The in vivo BLI performed a week after the last dose showed that bioluminescence signals were detected in 6 out of 7 (6/7), 6/8, 4/8, and 6/7 animals in the vehicle control, 1 mg/kg, 10 mg/kg and 50 mg/kg anti-GPC1mAb groups, respectively (Figure 8A). Tumor nodules were found in the lungs of two more animals in the 10 mg/kg anti-GPC1 mAb group during the macroscopic inspection. Animals that failed to develop macroscopic tumors in the lungs were excluded from further analyses. Results of the ELISA determination of the anti-GPC1 mAb levels in plasma on the final day of the experiment showed that the anti-GPC1 mAb plasma levels were strongly correlated with the administered dose (Pearson’s correlation coefficient = 0.9986, *P* < 0.01; Figure 8B). Tumor-bearing mice treated with 50 mg/kg anti-GPC1 mAb had 43-, 17- and 2-fold increase in the mean anti-GPC1 mAb plasma levels as compared with the control, 1 mg/kg and 10 mg/kg anti-GPC1 mAb groups, respectively (*P* < 0.01 for all; Figure 8B). The mean anti-GPC1 mAb level in the 10 mg/kg group was significantly higher than that in the control group (11-fold increase, *P* < 0.05; Figure 8B). These data indicated that i.p. administration of 5 doses of anti-GPC1 mAb at 10 mg/kg and 50 mg/kg over 41 days elicited significantly higher mAb plasma levels as compared with the vehicle control. With regard to the effect of anti-GPC1 mAb treatment on tumor burden, although the mean total lung weights in the 10 mg/kg and 50 mg/kg anti-GPC1 mAb groups were decreased by 28% and 14% as compared with those of the control group, the differences were not statistically significant (*P* > 0.05. Figure 8C).

### Evaluation of molecular changes in response to anti-GPC1 mAb treatment in orthotopic A549 NSCLC xenografts and adjacent normal lung tissues

**Figure 9:**
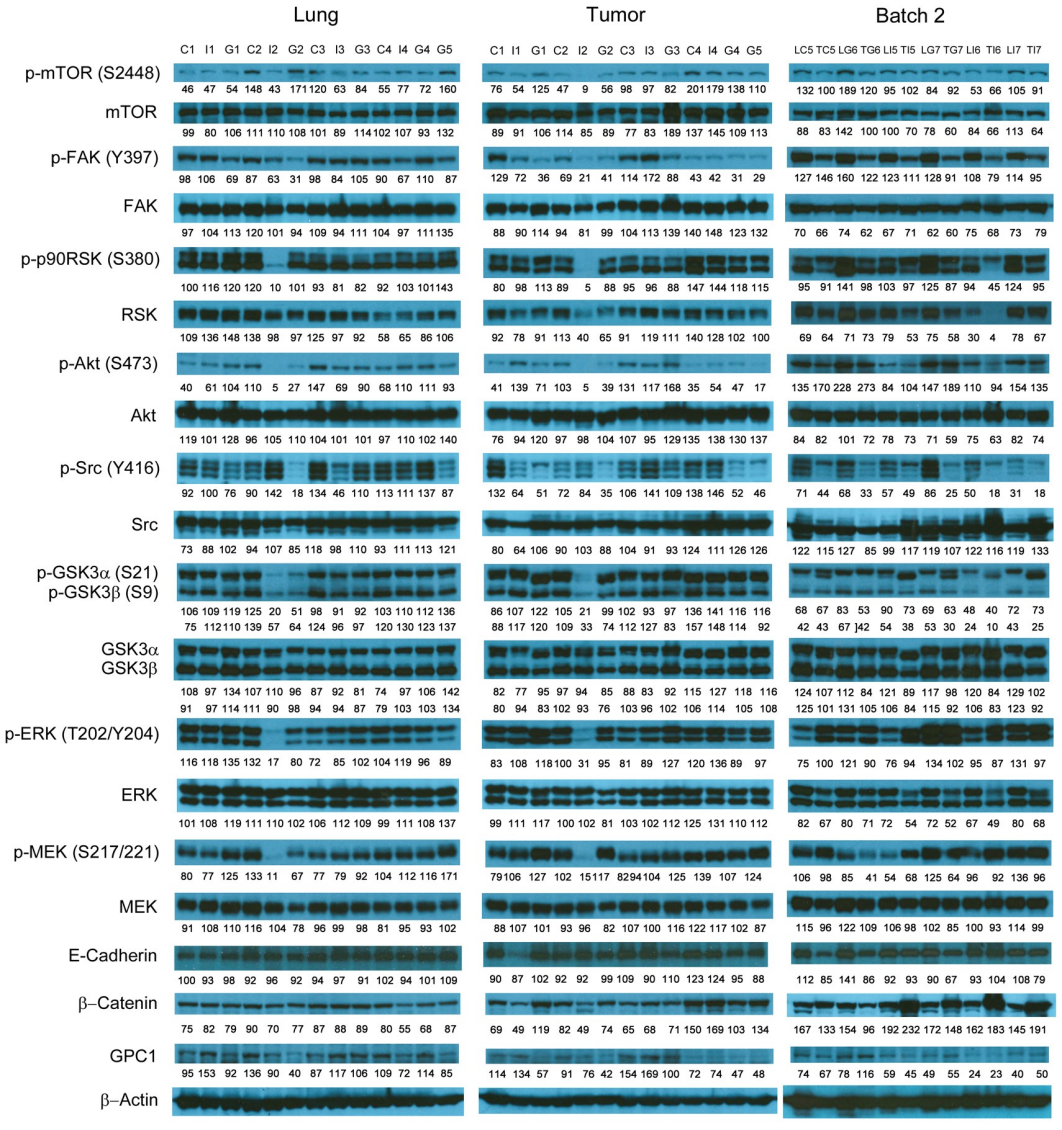
Western blot analysis of tumor and lung tissues obtained from the i.v. dosing study. Western blot images demonstrated the expression of total and phosphorylated mTOR, RSK1/2/3, FAK, Src Akt, GSK3α/3β, ERK1/2 and MEK1/2, selected EMT markers and GPC1 protein in individual tumor and lung tissue samples obtained for tumor bearing athymic nude mice treated with vehicle control, or 10 mg/kg mouse IgG isotype, or 10 mg/kg anti-GPC1 mAb through tail vein injection once every 72 h for 8 doses. Numbers below individual blots represent the percentage of the normalized integrated density values against β-actin (loading control). C: vehicle control; I: 10 mg/kg mouse IgG isotype; G: 10 mg/kg anti-GPC1 mAb; L: lung; T: tumor; p-FAK: phospho-focal adhesion kinase; p90RSK: 90 kDa ribosomal s6 kinase; GSK3α: glycogen synthase kinases 3α; ERK: extracellular signal-regulated kinase; MEK: mitogen-activated protein kinase kinase; GPC1: glypican-1

**Figure 10:**
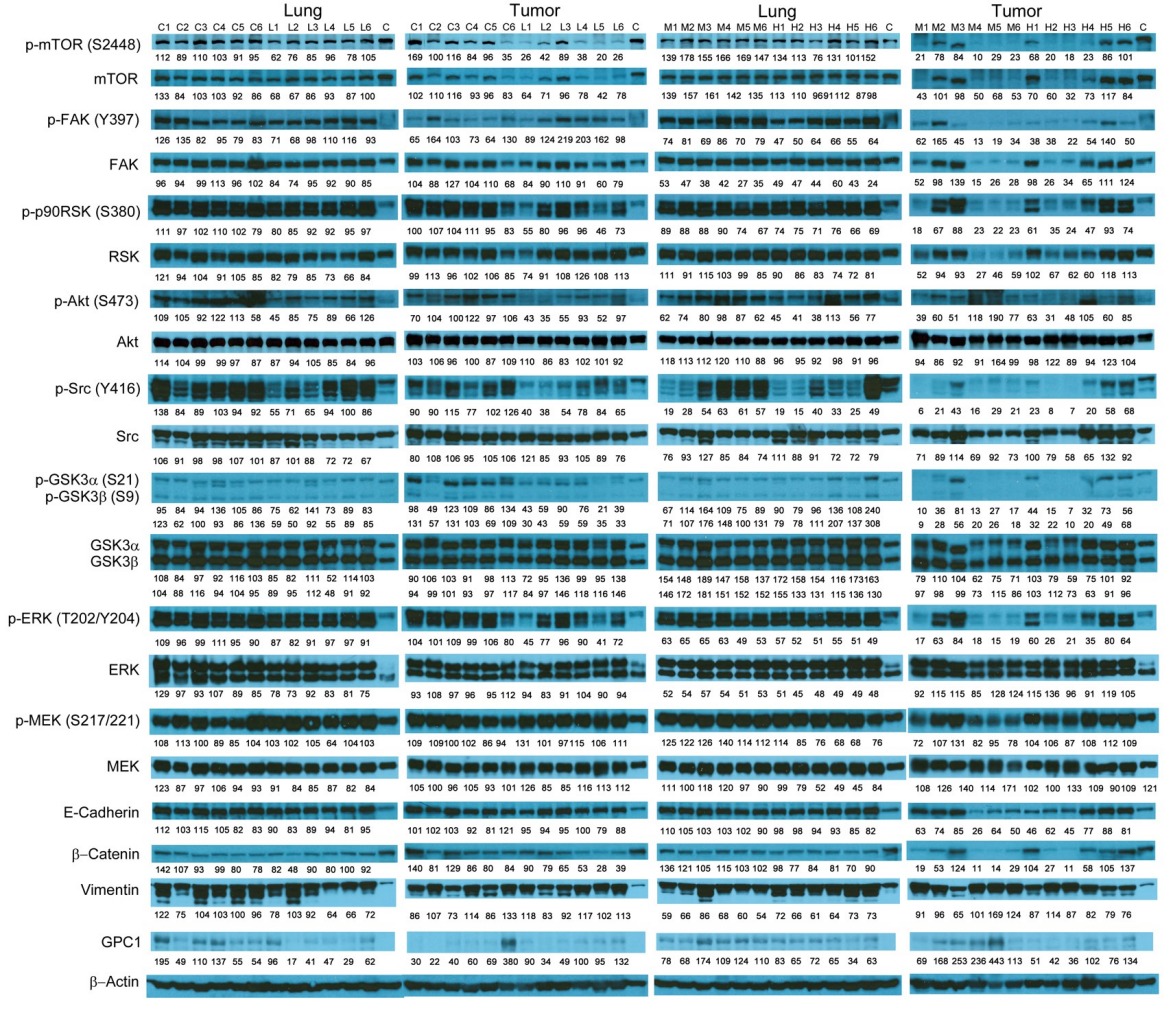
Western blot analysis of tumor and lung tissues obtained from the i.p. dosing study. Western blot images demonstrated the expression of total and phosphorylated mTOR, RSK1/2/3, FAK, Src, Akt, GSK3α/3β, ERK1/2 and MEK1/2, selected EMT markers and GPC1 protein in individual tumor and lung tissue samples obtained for tumor bearing athymic nude mice treated with vehicle control, 1 mg/kg, 10 mg/kg or 50 mg/kg of anti-GPC1 mAb through i.p. injection once a week for 3 doses and once every ten days for 2 more doses. Numbers below individual blots represent the percentage of the normalized integrated density values against β-actin (loading control). C: control; L: 1 mg/kg anti-GPC1 mAb; IPM: 10 mg/kg anti-GPC1 mAb; H: 50 mg/kg anti-GPC1 mAb. p-FAK: phospho-focal adhesion kinase; p90RSK: 90 kDa ribosomal s6 kinase; GSK3α: glycogen synthase kinases 3α; ERK: extracellular signal-regulated kinase; MEK: mitogen-activated protein kinase kinase; GPC1: glypican-1

**Figure 11:**
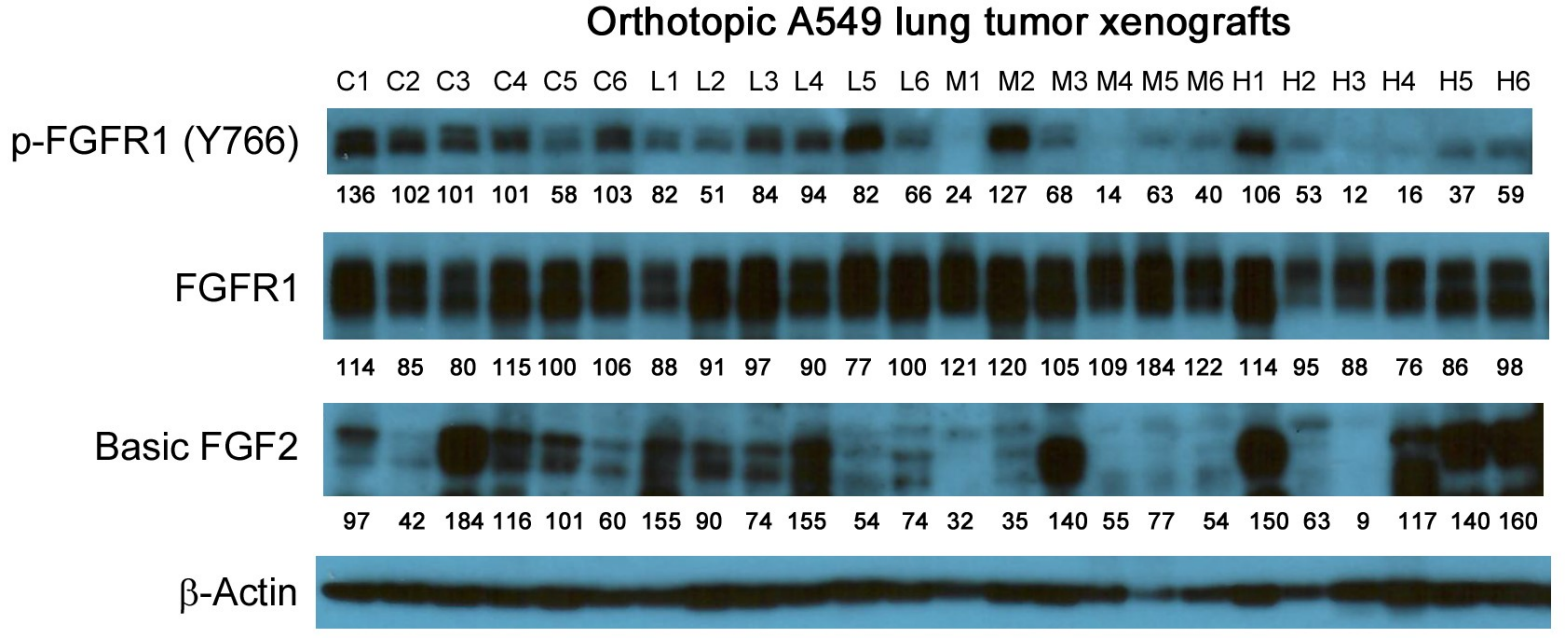
Western blot analysis of basic FGF2 and total and phosphorylated FGFR1 in A549 lung tumors. Individual tumor samples were obtained for tumor bearing athymic nude mice treated with vehicle control, 1 mg/kg, 10 mg/kg or 50 mg/kg of anti-GPC1 mAb through i.p. injection once a week for 3 doses and once every ten days for 2 more doses. Numbers below individual blots represent the percentage of the normalized integrated density values against β-actin (loading control). C: control; L: 1 mg/kg anti-GPC1 mAb; M: 10 mg/kg anti-GPC1 mAb; H: 50 mg/kg anti-GPC1 mAb. FGFR1: fibroblast growth factor receptor 1

**Figure 12:**
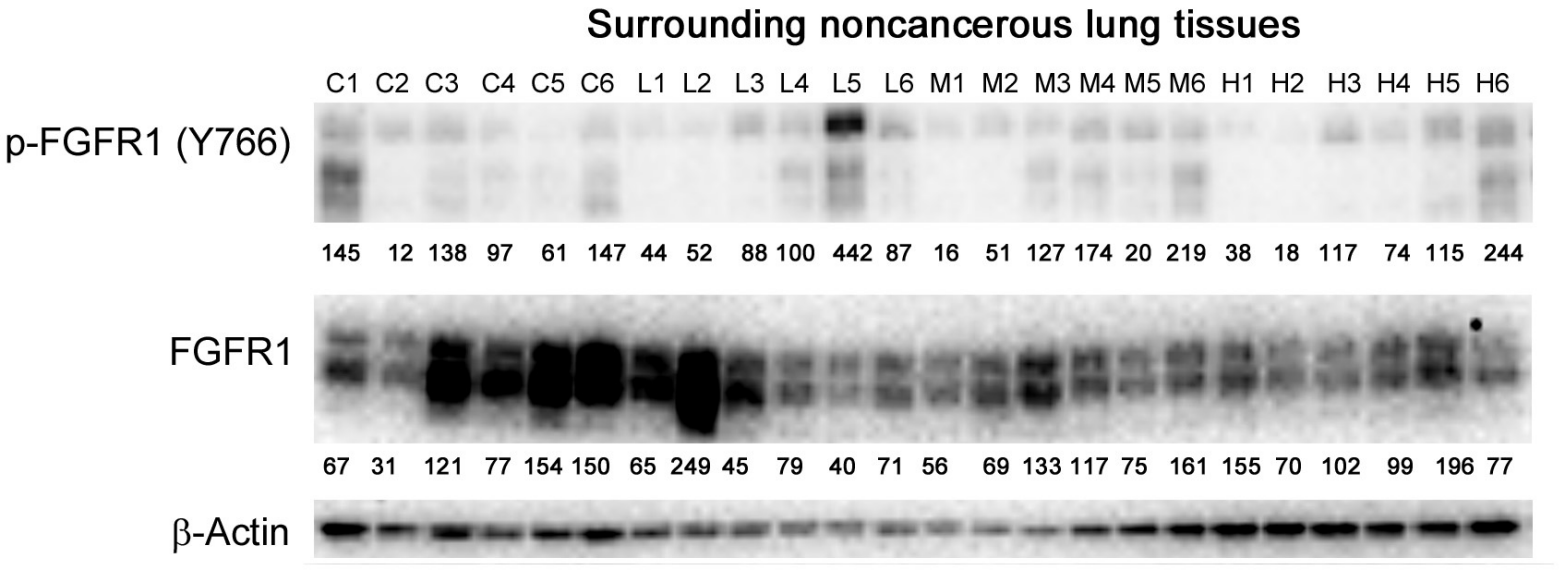
Western blot analysis of total and phosphorylated FGFR1 in adjacent noncancerous lung tissues. Individual lung tissue samples were obtained from tumor bearing athymic nude mice treated with vehicle control, 1 mg/kg, 10 mg/kg or 50 mg/kg of anti-GPC1 mAb through i.p. injection once a week for 3 doses and once every ten days for 2 more doses. Numbers below individual blots represent the percentage of the normalized integrated density values against β-actin (loading control). C: control; L: 1 mg/kg anti-GPC1 mAb; M: 10 mg/kg anti-GPC1 mAb; H: 50 mg/kg anti-GPC1 mAb. FGFR1: fibroblast growth factor receptor 1

**Figure 13:**
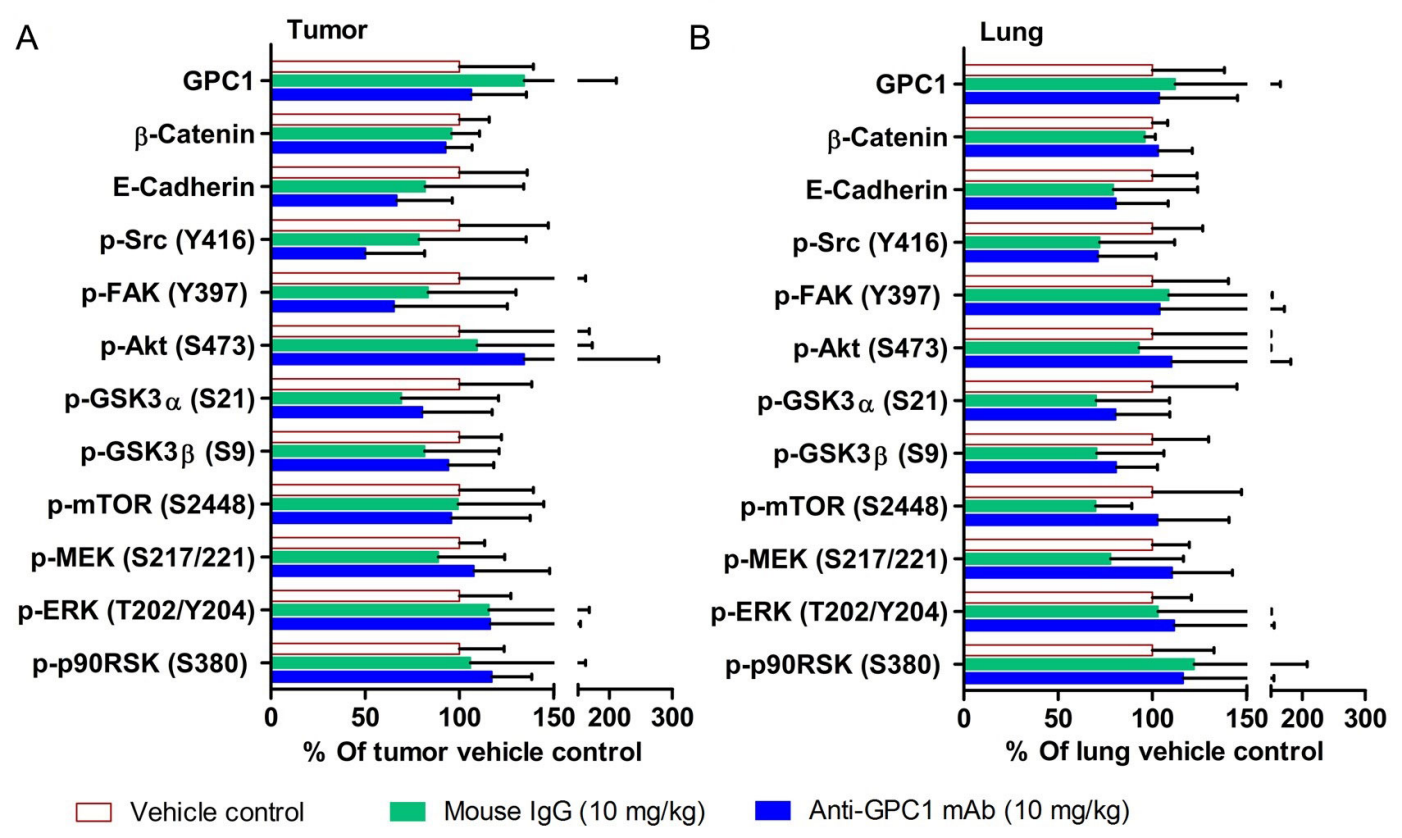
Semi-quantitative densitometric analysis of Western blot results obtained from the in vivo anti-GPC1 mAb i.v. dosing study. (A) A549 lung tumor xenografts; (B) adjacent noncancerous lung tissues. Tumor and adjacent noncancerous lung tissue samples were collected from athymic nude mice bearing established orthotopic A549 lung tumor and receiving i.v. administration of vehicle control (*n* = 5), 10 mg/kg mouse IgG isotype (*n* = 7), and 10 mg/kg anti-GPC1 mAb (*n* = 6) once every 72 h for a total of 8 doses. Tumor and lung tissue homogenate samples were subjected to Western blot analysis (80 μg of protein per lane). Detection of β-actin was used to ensure equal sample loading per lane. Relative immunoreactive band intensities are expressed as percent change over the average signal value in the vehicle-treated lung or tumor tissues. For phosphorylated proteins, results are expressed as the ratio of phosphorylated-to-total species relative to the vehicle control. Data are presented as mean ± SD. SD is denoted by the error bars. No statistically significant difference was found among the three study groups (*P* > 0.05). GPC1: glypican-1; p-FAK: phospho-focal adhesion kinase; GSK3α: glycogen synthase kinases 3α; MEK: mitogen-activated protein kinase kinase; ERK: extracellular signal-regulated kinase; p90RSK: 90 kDa ribosomal s6 kinase

The effect of in vivo anti-GPC1 mAb treatment on signaling pathways associated with aberrant activation of growth factor receptors, including the MEK/ERK/RSK, Akt/GSK3/mTOR and Src/FAK pathways, as well as the protein expression of selected EMT markers, was evaluated in A549 lung tumors and adjacent noncancerous lung tissues using Western blot analysis (Figure 9, Figure 10, Figure 11, and Figure 12). The i.v. administration of 10 mg/kg anti-GPC1 mAb once every 72 h for a total of 8 doses did not have any significant effect on the phosphorylation of MEK, ERK, RSK, Akt, GSK3α/β, mTOR, Src and FAK, nor did it change significantly the expression of E-cadherin and β-catenin protein in orthotopic A549 lung tumors and adjacent normal lung tissues (Figure 9 and Figure 13).

**Figure 14:**
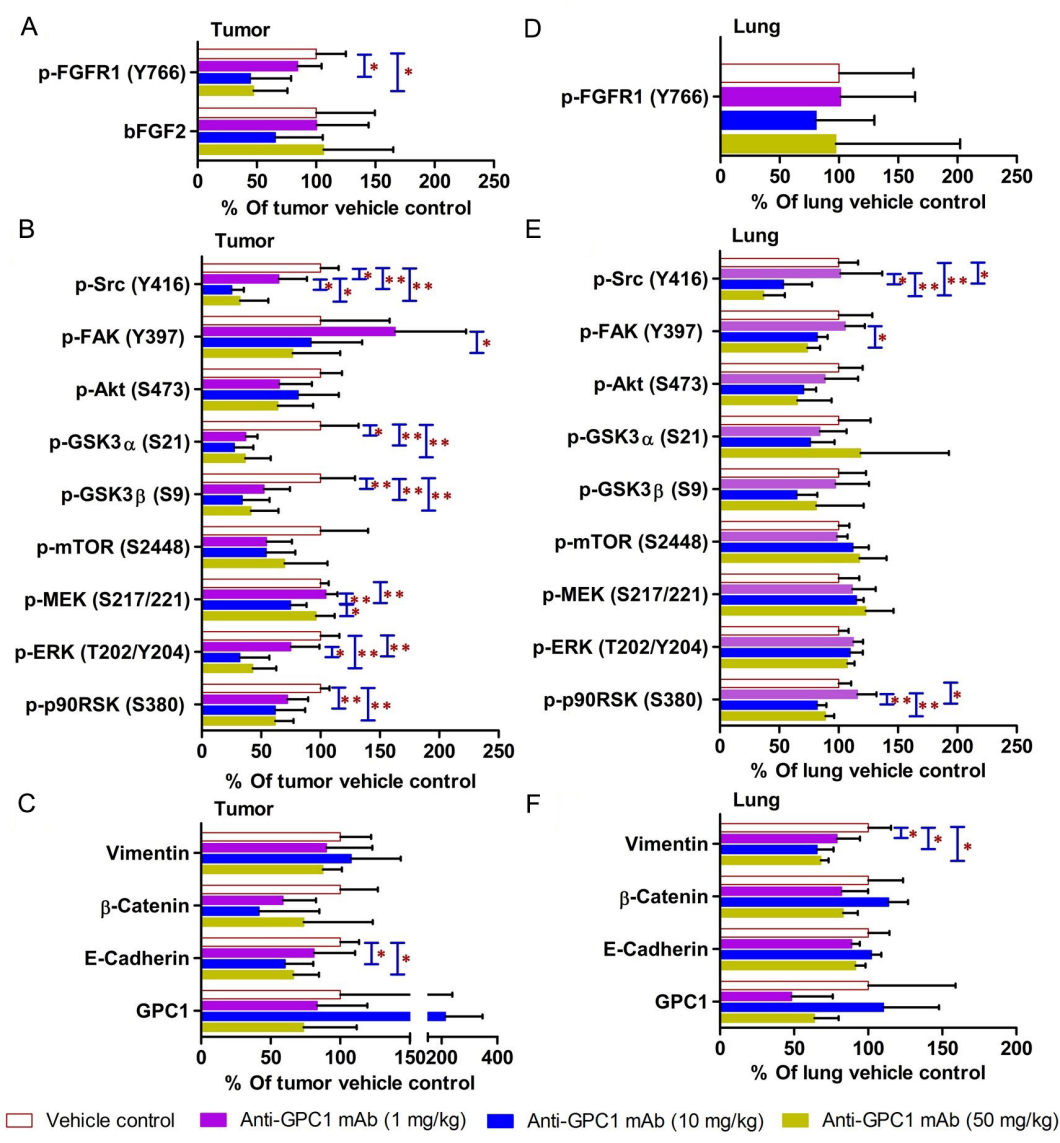
Semi-quantitative densitometric analysis Western blot results obtained from the in vivo anti-GPC1 mAb i.p. dosing study. (A) Expression of bFGF and p-FGFR1 in A549 lung tumors; (B) expression of effector proteins in MEK/ERK/RSK, Akt/GSK3/mTOR and Src/FAK pathways in A549 lung tumors; (C) expression of EMT markers in A549 lung tumors; (D) expression of p-FGFR1 in adjacent noncancerous lung tissues; (E) expression of effector proteins in MEK/ERK/RSK, Akt/GSK3/mTOR and Src/FAK pathways in adjacent noncancerous lung tissues; (F) expression of EMT markers in adjacent noncancerous lung tissues. Athymic nude mice received i.p. administration of vehicle control, 1 mg/kg, 10 mg/kg or 50 mg/kg of anti-GPC1 mAb two weeks after the orthotopic inoculation of A549 tumor cells. Each treatment was given to individual animals once a week for 3 doses and then once every 10 days for 2 more doses for a total of 8 doses. Tumor and lung tissue homogenate samples were subjected to Western blot analysis (80 μg of protein per lane). Detection of β-actin was used to ensure equal sample loading per lane. Relative immunoreactive band intensities are expressed as percent change over the average signal value in the vehicle-treated lung or tumor tissues. For phosphorylated proteins, results are expressed as the ratio of phosphorylated-to-total species relative to the vehicle control. Data are presented as mean ± SD (*n* = 6 for each study group). SD is denoted by the error bars. *P < 0.05 and **P < 0.01 compared among vehicle control, 1 mg/kg, 10 mg/kg and 50 mg/kg anti-GPC1 mAb groups using one-way ANOVA followed by Tukey-Kramer post hoc multiple comparison test. p-FGFR1: phospho-fibroblast growth factor receptor 1; bFGF2: basic fibroblast growth factor 2; FAK: focal adhesion kinase; GSK3α: glycogen synthase kinases 3α; MEK: mitogen-activated protein kinase kinase; ERK: extracellular signal-regulated kinase; p90RSK: 90 kDa ribosomal s6 kinase; GPC1: glypican-1

In contrast, the i.p. administration of anti-GPC1 mAb at 10 and 50 mg/kg once a week for 3 doses and then once every 10 days for 2 more doses appeared to attenuate the FGF signal transduction pathway in the orthotopic A549 NSCLC xenografts (Figure 10, Figure 11 and Figure 12). Treatment with 10 and 50 mg/kg anti-GPC1 mAb significantly inhibited the FGFR1 phosphorylation at Tyr766 (*P* < 0.05 for both treatment groups; Figure 11 and Figure 14A), as well as the downstream ERK phosphorylation at Thr202/Tyr204 and RSK phosphorylation at Ser380 in tumors, as compared with the control group (*P* < 0.01 for all; Figure 10 and Figure 14B). The relative expression levels of phospho-GSK3α (Ser21; *P* < 0.05 for 1 mg/kg, and *P* < 0.01 for 10 mg/kg and 50 mg/kg groups), phospho-GSK3β (Ser9; *P* < 0.01 for all) and phospho-Src (Tyr416; *P* < 0.05 for 1 mg/kg, and *P* < 0.01 for 10 mg/kg and 50 mg/kg groups) were significantly decreased in all anti-GPC1 mAb treated tumors as compared with those in the control group (Figure 10 and Figure 14B). Moreover, comparison among the treatment groups showed that the expression levels of phospho-Src (Tyr416) in A549 tumors treated with 10 mg/kg and 50 mg/kg anti-GPC1 mAb were significantly lower than those treated with 1 mg/kg anti-GPC1 mAb (*P* < 0.05 for both 10 mg/kg and 50 mg/kg groups; Figure 14B). The phospho-ERK (Thr202/Tyr204) expression in the 10 mg/kg group was significantly lower than that in the 1 mg/kg group (*P* < 0.05), while the phospho-MEK (Ser380) expression in the 10 mg/kg group was significantly lower than those in the 1 mg/kg (*P* < 0.01) and 50 mg/kg (*P* < 0.05) groups (Figure 14B). It was noted that treatment with anti-GPC1 mAb at 10 mg/kg and 50 mg/kg resulted in a significant decrease in the intratumoral E-cadherin protein expression as compared with the vehicle treatment (Figure 14C), raising the concern that anti-GPC1 mAb treatment might elicit EMT in tumors.

In adjacent noncancerous lung tissues, anti-GPC1 mAb had no effect on the phospho-FGFR1 (Tyr766) expression (*P* > 0.05; Figure 12 and Figure 14D). Anti-GPC1 mAb treatment at 10 mg/kg and 50 mg/kg significantly decreased the phospho-Src (Tyr416) expression as compared with the vehicle control (*P* < 0.05 and *P* < 0.01 for 10 mg/kg and 50 mg/kg anti-GPC1 mAb treatment, respectively) and the 1 mg/kg anti-GPC1 mAb treatment group (*P* < 0.05 and *P* < 0.01 for 10 mg/kg and 50 mg/kg anti-GPC1 mAb groups, respectively; Figure 10 and Figure 14E). Moreover, the phospho-p90RSK (Ser380) expression levels in the 10 mg/kg and 50 mg/kg anti-GPC1 mAb treatment groups were significantly lower than that in the 1 mg/kg group (*P* < 0.01 for both; Figure 14E). A significant difference in the phospho-p90RSK (Ser380) expression level in the noncancerous lung tissues was found between control and 10 mg/kg anti-GPC1 mAb groups (*P* < 0.05), while a significant difference in the phospho-FAK (Tyr397) expression levels was found between control and 10 mg/kg anti-GPC1 mAb treatment groups (*P* < 0.05; Figure 14E). It was noted that the protein expression of GPC1 was detectable using the anti-GPC1 mAb, which is developed to target human GPC1, suggesting that the anti-GPC1 mAb is cross-reactive with the mouse GPC1. Treatment with the anti-GPC1 mAb at all three dose levels significantly decreased the vimentin protein expression levels in the adjacent noncancerous lung tissues (*P* < 0.05 for all; Figure 14F), implicating that certain types of cells in normal lung tissue, such as lung fibroblasts, may be sensitive to anti-GPC1 mAb treatment.

## Discussion

The distinct role of GPC1 in promoting the progression of certain types of solid tumors has rendered it an attractive target for cancer therapy. Since inhibition of GPC1 activity with an anti-GPC1 mAb has shown promising anti-tumor effect in a GPC1-positive ESCC xenograft model, it is anticipated that GPC1 targeted therapies can be developed for other GPC1-positive solid tumors [29]. So far the therapeutic potential of selective inhibition of GPC1 for NSCLC treatment has not been documented, partly because of the unclear clinicopathological significance and diagnostic role of GPC1 in NSCLC [22, 23]. In order to verify the expression level of GPC1 mRNA in NSCLC, 10 public microarray datasets in Gene Expression Omnibus (series: GSE3268; accession number: GDS1312; gene probe: 202756_s_at) were analyzed. The calculated log2 transformed fold change of GPC1 mRNA levels indicated that GPC1 was up-regulated with 2.38-fold change in the squamous cell lung carcinoma biopsy specimens as compared with that in the paired normal lung tissues (adjusted *P*-value = 0.00399; Figure S1). Given the clinical relevance of GPC1 expression in NSCLC, this study was focused on evaluating the effect of a new anti-GPC1 mAb on lung tumor growth using in vitro and in vivo models and exploring the associated mechanisms.

In the in vitro cytotoxicity study, the anti-GPC1 mAb cytotoxicity was found in an ascending order with H460 cell monocultures (mean IC_50_ = 646 μg/mL) < A549 cell monocultures (mean IC_50_ = 169 μg/mL) < LL97A fibroblast monocultures (mean IC_50_ = 60 μg/mL), indicating that lung fibroblasts are more sensitive to the cytotoxic effect of anti-GPC1 mAb than NSCLC cells (Figure 1). Consistent with the finding obtained from monocultures, A549/LL97A and H460/LL97A coculture spheroids appeared to be more sensitive to the cytotoxic effect of anti-GPC1 mAb than A549 and H460 monoculture spheroids. Moreover, anti-GPC1 mAb was found to exhibit profound inhibitory effect on the anchorage-independent growth of A549 and H460 cells (Figure 2). Given that the anchorage-independency bestows upon tumor cells the potential to colonize distant organs and form malignant lesions [38], anti-GPC1 mAb treatment is likely to be effective against tumorigenesis and cancer metastasis. Furthermore, anti-GPC1 mAb treatment significantly inhibited the invasion potential of tumor cell/fibroblast coculture spheroids but not the tumor cell monoculture spheroids (Figure 3), suggesting that anti-GPC1 mAb suppresses fibroblast-led collective invasion of tumor cells by inhibiting the fibroblast activity [39].

Consistent with the results of in vitro cytotoxicity and invasion assays, the anti-GPC1 mAb treatment had little effect on the levels of phosphorylated signaling effectors that are major regulators of cell proliferation and survival in monocultured A549 and H460 cells (Figure 4 and Figure 5). In contrast, the anti-GPC1 mAb treatment significantly inhibited the phosphorylation of FAK at Tyr397, RSK at Ser380, Akt at Ser473 and mTOR at Ser2448 in monocultured LL97A lung fibroblasts, suggesting that the inhibitory effect of anti-GPC1 mAb on lung fibroblast proliferation is attributable to the deactivated Akt/mTOR pathway and reduced FAK and RSK activities (Figure 6A). However, when LL97A lung fibroblasts were co-cultured indirectly with A549 or H460 tumor cells, anti-GPC1 mAb significantly decreased the phosphorylation of Src at Tyr416 and Akt at Ser473 and the β-catenin protein expression level in LL97A fibroblasts (Figure 6B and 6C), and the expression of phospho-MEK (Ser217/221) and phospho-RSK (Ser380) in co-cultured A549 cells (Figure 6D). The difference in molecular response to anti-GPC1 mAb treatment between monocultured and co-cultured lung fibroblasts and tumor cells implicates that anti-GPC1 mAb impairs the reciprocal crosstalk between tumor cells and tumor-associated fibroblasts.

Since the mean IC_50_ value of anti-GPC1 mAb in A549 cells determined after 72-hour treatment in vitro was 0.169 mg/mL, and given the fact that an adult male mouse usually has a circulating blood volume of about 58.5 mL/kg body weight (https://nc3rs.org.uk/mouse-decision-tree-blood-sampling), athymic nude mice with established orthotopic A549 lung tumors were given the i.v. administration of the anti-GPC1 mAb at a dose of 10 mg/kg once every 72 h for eight doses in the initial in vivo study (0.169 mg/mL × 58.5 mL/kg = 9.9 mg/kg ≈ 10 mg/kg). Although the anti-GPC1 mAb treatment reduced the mean relative rate of lung tumor growth, the difference was not statistically different between the anti-GPC1 mAb treatment group and the control groups (including both vehicle and 10 mg/kg mouse IgG isotype groups; *P* > 0.05; Figure 7B). Consistent with the relative lung tumor growth rate data, the mean total lung weight in the anti-GPC1 mAb group was not significantly different from those in the vehicle and mouse IgG isotype groups (Figure 7C). Moreover, results of the Western blot analysis of tumor samples indicated that the short-term anti-GPC1 mAb treatment had little effect on the activity of the MEK/ERK/RSK and Akt/GSK3/mTOR pathways (Figure 9 and Figure 13). Although the anti-GPC1 mAb treatment appeared to reduce the FAK Tyr397 and Src Ser416 phosphorylation in tumors by 34% and 52%, respectively, as compared with the vehicle control treatment, the difference was not statistically significant due to large interindividual variability (Figure 13A). The lack of anti-tumor effect of the short-term anti-GPC1 mAb treatment against the established orthotopic A549 lung tumors and the profound inhibitory effect of anti-GPC1 mAb on anchorage-independent A549 cell growth in vitro suggest that the effect of anti-GPC1 mAb on preventing tumor development in tumor-bearing mice should be tested using a modified anti-GPC1 mAb dosing regimen.

**Figure 15:**
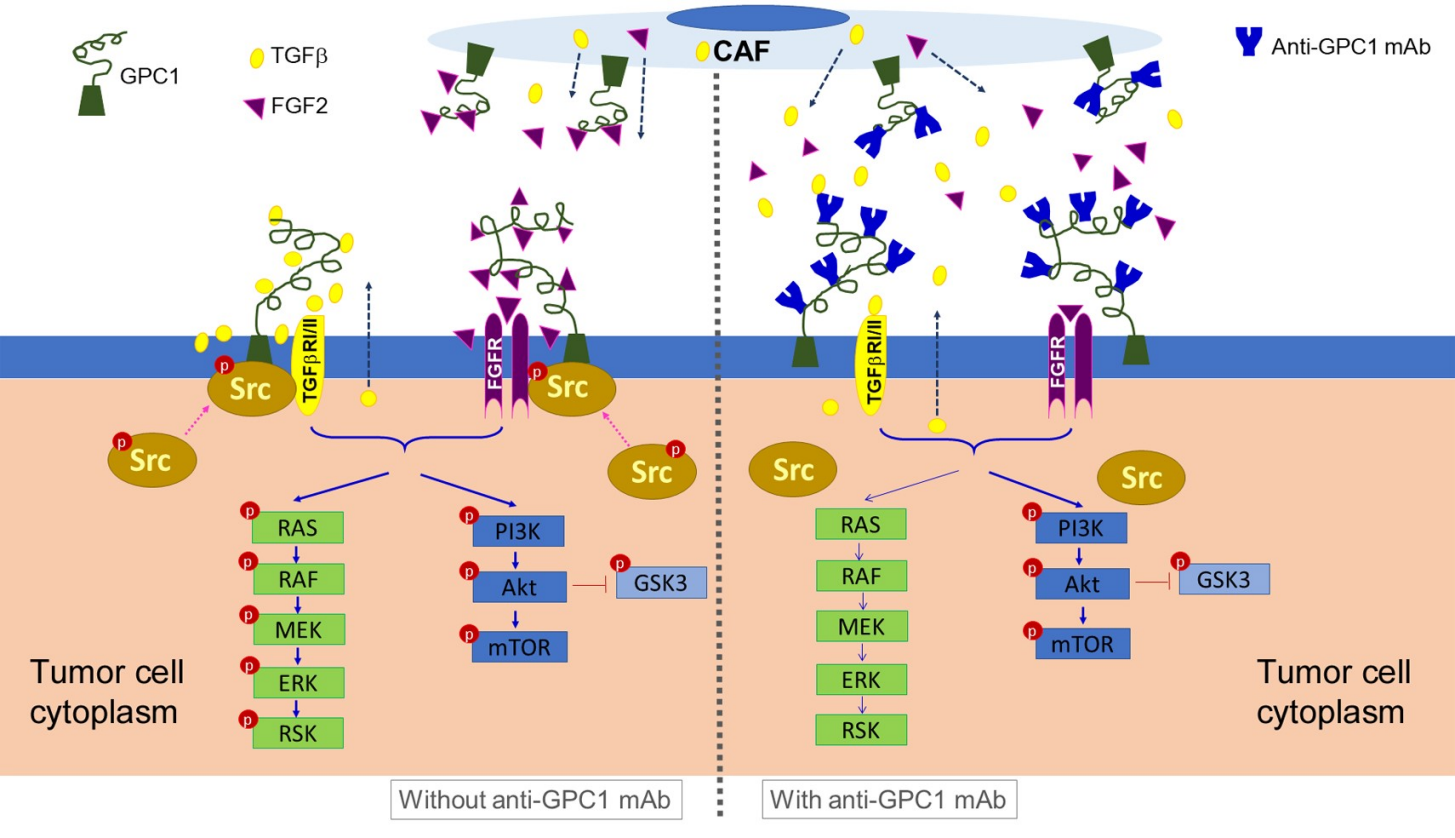
Schematic presentation of the proposed mechanism underlying the anti-tumor activity of anti-GPC1 mAb. It is postulated that anti-GPC1 mAb impairs the GPC1-facilitated encounter of growth factors with receptors that requires the Y416 phosphorylation of Src and recruitment of active Src onto the plasma membrane to initiate the signal transduction, leading to the attenuation of downstream MEK/ERK/RSK signaling pathway. CAF: cancer-associated fibroblast; GPC1: glypican-1; TGFβ: tumor growth factor β; mAb: monoclonal antibody; FGF2: fibroblast growth factor 2; FGFR: fibroblast growth factor receptor; RAS: rat sarcoma virus; RAF: rapidly accelerated fibrosarcoma; MEK: mitogen-activated protein kinase kinase; ERK: extracellular signal-regulated kinase; RSK: ribosomal s6 kinase; GSK3: glycogen synthase kinases 3

In the second in vivo study, the anti-GPC1 mAb treatment was started two weeks after the orthotopic inoculation of A549 tumor cells for early blockage of GPC1 activity. The dosage regimen for anti-GPC1 mAb was modified to i.p. administration of the mAb at dose levels of 1 mg/kg, 10 mg/kg and 50 mg/kg once a week for 3 doses and then once every 10 days for 2 more doses. With the modified dosage regimen, the anti-GPC1 mAb plasma levels determined in the 10 mg/kg and 50 mg/kg groups seven days after the last dose were 11- and 43-fold, respectively, higher than that in the vehicle control group (*P* < 0.05 and *P* < 0.01, respectively; Figure 8B), implicating the slow clearance and long half-life of the anti-GPC1 mAb [40]. Although the modified anti-GPC1 mAb dosage regimen was unable to reduce the lung tumor burden significantly as reflected by the total lung weight (Figure 8C), the anti-GPC1 mAb treatment significantly inhibited the FGFR1 phosphorylation at Tyr766 in tumors without affecting the FGF2 levels in tumors and subsequently reduced the phosphorylation of several prominent effectors downstream of the FGFR1, including ERK [41], RSK, GSK3α and GSK3β (Figure 10, Figure 11, Figure 12, and Figure 14). The possible mechanism underlying the inhibitory effect of the anti-GPC1 mAb on the FGFR1 signaling is two-fold. On the one hand, since GPC1 is known to act as a co-receptor by sequestering the FGFs on the cell surface, stabilizing the FGF ligand-receptor interaction and subsequently improving the efficiency of FGF-activated FGFR1 signaling pathways in tumor cells [5, 6, 42], it is conceivable that inhibition of GPC1 activity with the anti-GPC1 mAb is able to impede the activation of signaling pathways downstream of FGFR1. In this study, it appeared that the MEK/ERK/RSK signaling pathway was affected by the GPC1 inhibition more significantly than the Akt/mTOR signaling pathway in A549 orthotopic lung tumors, and inhibition of the MEK/ERK/RSK signaling was associated with the decreased phosphorylation of GSK3α and GSK3β [43, 44] (Figure 15). This proposed mechanism was in line with the in vitro finding that anti-GPC1 mAb treatment significantly decreased the expression of phospho-MEK and phospho-p90RSK in A549 cells that were cultured in indirect co-culture with the LL97A lung fibroblasts (*P* < 0.05 for both as compared with the vehicle control; Figure 6D). On the other hand, since tumor-associated fibroblasts can secrete high amounts of growth factors, such as TGFβ, HGF and FGF, to stimulate tumor progression [45], it is postulated that anti-GPC1 mAb treatment reduces the production of those growth factors by inhibiting the proliferation and activities of tumor-associated fibroblasts. This hypothesis was supported by the in vitro data of this study, which indicated that the lung fibroblasts were more sensitive to the cytotoxic effect of anti-GPC1 mAb as compared with the A549 and H460 lung tumor cells (Figure 1 and Figure 6).

Results of the initial in vivo study showed that short-term anti-GPC1 mAb treatment resulted in 52% decrease in Src phosphorylation at Tyr416 in tumors as compared with the control (Figure 13A). However, the difference was not statistically significant. Results of the second in vivo study, in which i.p. administration of anti-GPC1 mAb at a modified dosing regimen was started two weeks after tumor cell inoculation, demonstrated that anti-GPC1 mAb treatment at 1 mg/kg, 10 mg/kg and 50 mg/kg significantly reduced the phospho-Src (Tyr416) levels in A549 tumors by 35% (*P* < 0.05), 75% (*P* < 0.01) and 68% (*P* < 0.01), respectively (Figure 14B). Src is a cytoplasmic nonreceptor tyrosine kinase involved in multiple signaling pathways [46]. It is evident that Src can be constitutively associated with or recruited onto the cholesterol/sphingolipids-enriched microdomains in plasma membrane, also known as “lipid rafts”, to coordinate receptor signal transduction [47, 48]. It has been reported that the active Src is recruited to the FGF2-activated FGFR1 located at the plasma membrane to influence FGFR1 signaling dynamics through the adaptor protein fibroblast growth factor receptor substrate 2 (FRS2) [49, 50]. FRS2 is a lipid raft-associated protein serving as the primary link between FGFR and a variety of downstream pathways [51]. Without FGF2 stimulation, Src stays inactive in a cytoplasmic and tight perinuclear position showing undetectable Tyr416 phosphorylation [50]. In this regard, anti-GPC1 mAb may attenuate the binding affinity of FGF2 for FGFR1 and impede the activation of FGFR1 and subsequent recruitment of active Src onto the plasma membrane (Figure 15). Other GPC1-facilitated growth factor encounters with receptor signaling may also require the Y416 phosphorylation of Src and recruitment of active Src onto the cell membrane. For example, Cripto-1 (CR-1), a member of the epidermal growth factor-Cripto/FRL-1/Cryptic (EGF-CFC) gene family with the GPI anchorage domain (Ser161-Try188) attached to lipid rafts [52, 53], has been shown to specifically bind to GPC1 and subsequently activate the cytoplasmic Src [54]. Given that CR-1 is often expressed at high levels in NSCLC [55, 56], it is possible that the anti-GPC1 mAb treatment hinders the interaction between CR-1 and its cognate receptors, thereby alleviating the need for recruiting the phosphorylated Src in cytoplasm.

In the in vitro study, results of the Western blotting revealed that the anti-GPC1 mAb treatment significantly decreased the vimentin protein expression levels in cultured LL97A fibroblasts that do not express E-cadherin protein at detectable levels (data not shown), while the vimentin and E-cadherin protein expression levels in A549 and H460 cells were not affected by the treatment (Figure 6), suggesting that the inhibitory effect of anti-GPC1 mAb on tumor cell invasion is not associated with changes in the EMT process of tumor cells but the attenuated activities of tumor-associated fibroblasts. In the in vivo study, the vimentin protein expression in normal lung tissues was significantly decreased in all anti-GPC1 mAb treatment groups, while the E-cadherin protein expression in A549 lung tumors was significantly decreased in 10 mg/kg and 50 mg/kg anti-GPC1 mAb treatment groups (Figure 14). Since vimentin is required for early-stage lung adenocarcinoma dissemination by acting as a regulator of the motility of tumor-associated fibroblasts [57], the observed decrease in vimentin protein expression in anti-GPC1 mAb-treated LL97A lung fibroblast monoculture and in mouse lung tissues implicates that the anti-GPC1 mAb may inhibit the fibroblasts-led collective invasion of tumor cells [58]. Although it has been reported that inhibition of Src activity led to the inhibition of E-cadherin removal from cell membrane and lysosomal degradation [59, 60], the exact mechanism involved in the decreased E-cadherin protein level in anti-GPC1 mAb treated A549 tumors remains unclear.

Taken together, the data of this study implicate that the antitumor potential of anti-GPC1 mAb lies in its ability to impede the GPC1-facilitated encounter of FGF2 with FGFR that requires the Y416 phosphorylation of Src to initiate the signal transduction, and subsequently attenuate the downstream MEK/ERK/RSK signaling pathway (Figure 15). This finding offers new insight into the molecular response to anti-GPC1 mAb treatment in NSCLC, providing a rational basis for a combination strategy that incorporates a GPC1 inhibitor to sensitize tumors to certain targeted therapeutics. In addition, given the evidence of the cultured lung fibroblasts being more sensitive to anti-GPC1 mAb treatment than NSCLC cells, and the significantly decreased vimentin protein expression in anti-GPC1 mAb-treated LL97A lung fibroblast monocultures and mouse lung tissues, it is postulated that selective inhibition of GPC1 may reduce the fibroblasts-led collective invasion of tumor cells. Further study is needed to investigate the impact of selective inhibition of GPC1 with anti-GPC1 mAb on the crosstalk between tumor cells and tumor-associated fibroblasts in NSCLC.

## Abbreviations

BLI: bioluminescence imaging
CR-1: Cripto-1
DMEM: Dulbecco’s Modified Eagle’s Medium
EGF-CFC: epidermal growth factor-Cripto/FRL-1/Cryptic
ELISA: enzyme-linked immunosorbent assay
EMT: epithelial-mesenchymal transition
ERK: extracellular signal-regulated kinase
ESCC: esophageal squamous cell carcinoma
FAK: focal adhesion kinase
FBS: fetal bovine serum
FGF2: fibroblast growth factor 2
FGFR1: fibroblast growth factor receptor 1
FRS2: fibroblast growth factor receptor substrate 2
GFR: growth factor reduced
GPC1: glypican-1
GPI: glycosylphosphatidylinositol
GSK3α: glycogen synthase kinases 3α
HB: heparin-binding
HGF: hepatocyte growth factor
HHs: Hedgehogs
i.p.: intraperitoneal
i.v.: intravascular
IACUC: institutional animal care and use committee
IgG: immunoglobulin G
mAb: monoclonal antibody
MEK: mitogen-activated protein kinase kinase
NSCLC: non-small cell lung carcinoma
PBS: phosphate buffered saline
PBST: phosphate-buffered saline containing 0.1% Tween 20
RSK: 90 kDa ribosomal S6 kinase
SD: standard deviation
TGFβ: tumor growth factor β
VEGF: vascular endothelial growth factors
Wnt: Wingless-type

## Supplementary materials

The supplementary material for this article is available at: https://www.explorationpub.com/uploads/Article/file/1002238_sup_1.pdf.

## Declarations

### Availability of data and materials

The raw data supporting the conclusions of this manuscript will be made available upon request from the corresponding authors (ccao@usf.edu; qzhou1@usf.edu), without undue reservation, to any qualified researchers.

### Copyright

© The Author(s) 2024.
