# Supplementary material for: Evaluation of antitumor potential of an anti-glypican-1 monoclonal antibody in preclinical lung cancer models reveals a distinct mechanism of action
Source: Explor Target Antitumor Ther. 2024 Jun 17;5(3):600–26. doi: 10.37349/etat.2024.00238 (PMC11220310; doi:10.37349/etat.2024.00238)
Supplement: Supplementary file 1 [file 1002238_sup_1.pdf]

GPC1 full protein sequence (1–559)

MELRARGWWLLCAAAALVACARGDPASKSRSCGEVRQIYGAKGFSLSDVPQAEISGEH
LRICPQGYTCCTSEMEENLANRSHAELETALRDSSRVLQAMLATQLRSFDDHFQHLLND
SERTLQATFPGAFGELYTQNARAFRDLYSELRLYYRGANLHLEETLAEFWARLLERLFK
QLHPQLLLPDDYLDCLGKQAEALRPFGEAPRELRLRATRAFVAARSFVQGLGVASDVV
RKVAQVPLGPECSRAVMKLVYCAHCLGVPGARPCPDYCRNVLKGCLANQADLDAEW
RNLLDSMVLITDKFWGTSGVESVIGSVHTWLAEAINALQDNRDTLTAKVIQGCGNPKV
NPQGPGPEEKRRRGKLAPRERPPSGTLEKLVSEAKAQLRDVQDFWISLPGTLCSEKMAL
STASDDRCWNGMARGRYLPEVMGDGLANQINNPEVEVDITKPDMTIRQQIMQLKIMTN
RLRSAYNGNDVDFQDASDDGSGSGSGDGCLDDLCSRKVSRKSSSSRTPLTHALPGLSEQ
EGQKTSAASCPQPPTFLLPLLLFLALTVARPRWR

GPC1 1–280 protein sequence

MELRARGWWLLCAAAALVACARGDPASKSRSCGEVRQIYGAKGFSLSDVPQAEISGEH
LRICPQGYTCCTSEMEENLANRSHAELETALRDSSRVLQAMLATQLRSFDDHFQHLLND
SERTLQATFPGAFGELYTQNARAFRDLYSELRLYYRGANLHLEETLAEFWARLLERLFK
QLHPQLLLPDDYLDCLGKQAEALRPFGEAPRELRLRATRAFVAARSFVQGLGVASDVV
RKVAQVPLGPECSRAVMKLVYCAHCLGVPGARPCPDYCRNVLKGCL

GPC1 240–559 protein sequence

VPLGPECSRAVMKLVYCAHCLGVPGARPCPDYCRNVLKGCLANQADLDAEWRNLLDS
MVLITDKFWGTSGVESVIGSVHTWLAEAINALQDNRDTLTAKVIQGCGNPKVNPQGPGP
EEKRRRGKLAPRERPPSGTLEKLVSEAKAQLRDVQDFWISLPGTLCSEKMALSTASDDR
CWNGMARGRYLPEVMGDGLANQINNPEVEVDITKPDMTIRQQIMQLKIMTNRLRSAYN
GNDVDFQDASDDGSGSGSGDGCLDDLCSRKVSRKSSSSRTPLTHALPGLSEQEGQKTSA
ASCPQPPTFLLPLLLFLALTVARPRWR

Table S1. Primary antibodies used in western blot analyses

Protein	Molecular size (kDa)	Reactivity	Manufacturer	Catalog number
Akt	60	H, M, R, Mk, Dm	Cell Signaling Technology	4691
Phospho-Akt (Ser473)	60	H, M, R, Hm	Cell Signaling Technology	4051
ERK1/2	42, 44	H, M, R, Hm, Mk	Cell Signaling Technology	4695
Phospho-ERK1/2 (Thr202/Tyr204)	42, 44	H, M, R, Hm, Mk	Cell Signaling Technology	4370
GSK3α/3β	51 (GSK3α), 46 (GSK3β)	H, M, R, Hm, Mk	Cell Signaling Technology	5676
Phospho-GSK3α/3β (Ser21/Ser9)	51, 46	H, M, R, Mk, Z	Cell Signaling Technology	9331

MEK1/2	42	H, M, R, Mk, Dm	Cell Signaling Technology	8727
Phospho-MEK1/2 (Ser217/221)	42	H, M, R, Mk	Cell Signaling Technology	9154
mTOR	289	H, M, R, Mk	Cell Signaling Technology	4517
Phospho-mTOR (Ser2448)	289	H, M, R, Mk	Cell Signaling Technology	2971
RSK1/2/3	90	H, M, R, Mk, GP	Cell Signaling Technology	9355
Phospho-p90RSK (Ser380)	90	H, M, R, Mk, Mi	Cell Signaling Technology	11989
GPC1	62	H, M	MegaNano Biotech Inc.	N/A
Phospho-Src (Tyr416)	60	H, M, R, Mk	Cell Signaling Technology	6943
Src	60	H, M, R, Mk	Cell Signaling Technology	2108
Phospho-FAK (Tyr397)	125	H, M, R, Hm, Pg	Cell Signaling Technology	3283
FAK	125	H, M	Cell Signaling Technology	13009
Basic FGF2	18, 22, 24	H	Cell Signaling Technology	20102
FGFR1	92, 120, 145	H, M, R, Mk	Cell Signaling Technology	9740
Phospho-FGFR1 (Tyr766)	120, 145	H	Cell Signaling Technology	2544
E-Cadherin	130	H, M	Cell Signaling Technology	3195
β-Catenin	92	H, M, R, Mk	Cell Signaling Technology	8480
Vimentin	50, 57	H, M, R, Mk	Cell Signaling Technology	5741
β-Actin	42	H, M, R, Hm, Mk, Dg	Cell Signaling Technology	3700

Note: Individual primary antibodies were diluted as 1:1000 in TBST containing 5% w/v BSA

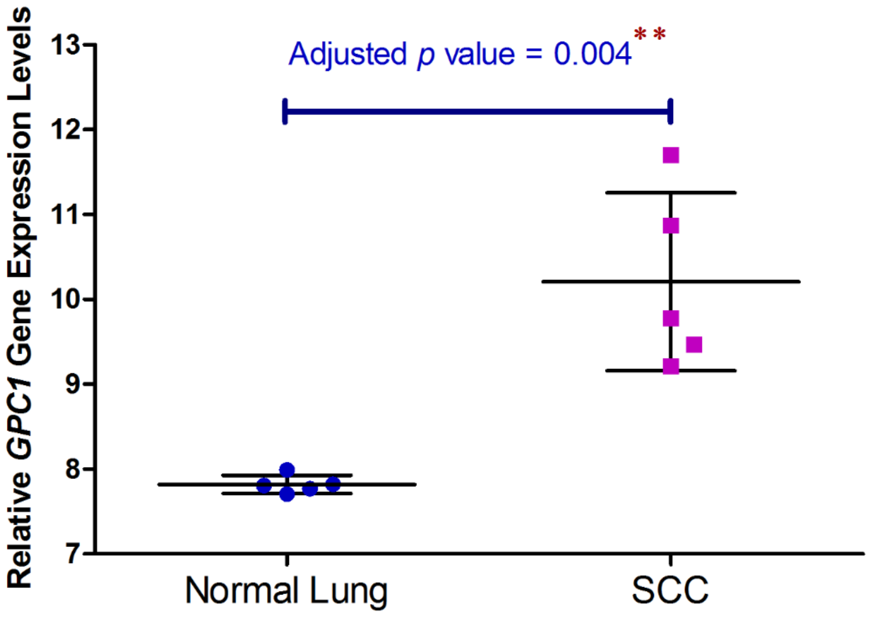

Figure S1. *GPC1* Log 2 transformed gene expression level. Relative *GPC1* gene expression values obtained from the GEO dataset GSE 3268 (accession number: GDS1312; gene probe: 202756_s_at) which contains expression profiling of squamous cell lung carcinoma (SCC) biopsy specimens and paired normal specimens form 5 patients
